# Differential ratio amplicons (*R*_amp_) for the evaluation of RNA integrity extracted from complex environmental samples

**DOI:** 10.1111/1462-2920.14516

**Published:** 2019-02-12

**Authors:** Fabien Cholet, Umer Z. Ijaz, Cindy J. Smith

**Affiliations:** ^1^ Infrastructure and Environment Research Division School of Engineering, University of Glasgow Glasgow, G12 8LT UK

## Abstract

Reliability and reproducibility of transcriptomics‐based studies are dependent on RNA integrity. In microbial ecology, microfluidics‐based techniques, such as the Ribosomal Integrity Number (RIN), targeting rRNA are currently the only approaches to evaluate RNA integrity. However, the relationship between rRNA and mRNA integrity is unknown. Here, we present an integrity index, the Ratio Amplicon, *R*_amp_, adapted from human clinical studies, to directly monitor mRNA integrity from complex environmental samples. We show, in a suite of experimental degradations of RNA extracted from sediment, that while the RIN generally reflected the degradation status of RNA the *R*_amp_ mapped mRNA degradation better. Furthermore, we examined the effect of degradation on transcript community structure by amplicon sequencing of *16S rRNA*, *amoA* and *glnA* transcripts. We successfully sequenced transcripts for all three targets even from highly‐degraded RNA samples. While RNA degradation changed the community structure of the mRNA profiles, no changes were observed for the 16S rRNA transcript profiles. Since both RT‐Q‐PCR and sequencing results were obtained, even from highly degraded samples, we strongly recommend evaluating RNA integrity prior to downstream processing to ensure meaningful results. For this, both the RIN and *R*_amp_ are useful, with the *R*_amp_ better evaluating mRNA integrity in this study.

## Introduction

A key question in environmental microbiology is to determine the functioning and activity of microbial communities. While genomic approaches have resulted in an unprecedented understanding of their structure and complexity (Medini *et al*., 2008), they do not inform of the activity and functioning at a given time. In this case, targeting the transcriptome, that is the subset of genes that are actively transcribed at a given time, is more informative. While there can be substantial post‐translational regulation that may prevent final protein synthesis and/or activity, gene expression is the direct link between the genome and the function it encodes and, therefore, a stronger link to activity than DNA approaches alone (Moran *et al*., 2013). As a result, transcriptomics‐based approaches are widely used to assess microbial activity and functioning in the environment (Smith *et al*., 2006; Evans, 2015). The premise is that messenger RNA (mRNA) turn‐over within cells is rapid, ranging from a few minutes to less than an hour (Laalami *et al*., 2014). As such, a snap‐shot of the transcriptome reflects the cells transcriptional response to its surrounding environment and metabolic needs at a given time.

A challenge for all transcript‐based studies, not least for those from environmental samples, is to ensure the quality and integrity of the RNA on which the results are based. Extracted RNA is prone to degradation both during the extraction procedure, post‐extraction handling and over time. Factors such as *RNase* activity, physical degradation during extraction procedures and even storage can degrade RNA. If there is significant post‐extraction degradation among different samples that are to be compared, the interpretation of results may be compromised. In other words, differences between samples may arise as a result of post‐extraction degradation, as opposed to representing actual difference in gene expression. Indeed, meaningful and reproducible results can only be obtained when working with good quality, intact RNA, whether it is eukaryotic RNA (Fleige and Pfaffl, 2006; Fleige *et al*., 2006; Copois *et al*., 2007; Die and Román, 2012) or Prokaryotic RNA (Jahn *et al*., 2008). As such an initial quality check of extracted RNA, not least from complex environmental microbial communities should be the essential first step before proceeding to any downstream applications. This quality check would help to ensure that any differences observed between samples are due to actual changes in gene expression rather than differences in samples integrity as a result of degradation.

In microbial ecology, current methods to evaluate the integrity of extracted RNA are based on ribosomal RNA (rRNA). These approaches evaluate integrity as a ratio between the 23S and 16S ribosomal RNA: 23S, 16S and 5S rRNA are synthetized as one primary transcript and are separated upon maturation (Kaczanowska and Ryden‐Aulin, 2007). The 23S and 16S ribosome should therefore be present at a ratio 1:1. However, as the 23S ribosome is approximately twice as long as the 16S ribosome, for intact, non‐degraded RNA, the expected ratio of 23S:16S RNA is 2:1. However, the caveat of this approach is the assumption that the integrity of rRNA reflects that of the overall RNA, including mRNA. The relationship between the integrity of rRNA and that of mRNA has not been demonstrated (Die and Román, 2012). Indeed, the formation of secondary structures and the interaction with ribosomal proteins may help protect ribosomes from degradation and could explain the more stable properties of rRNA compared with mRNA (Bonincontro *et al*., 1998; McKillip *et al*., 1998; 1999; Fontaine and Guillot, 2003; Rathnayaka and Rakshit, 2010; Rhodes *et al*., 2012; Sunyer‐Figueres *et al*., 2018). As such, the usefulness of this ratio to assess mRNA integrity is still unclear.

In its simplest form, evaluating ribosomal RNA integrity is an electrophoretic separation of RNA in a gel matrix. Essentially, a visual check for the presence of the characteristic bands corresponding to 23S and 16S rRNA. More advanced techniques based on microfluidics are better suited for assessing RNA quality, allowing for the calculation of integrity indexes, such as the RNA Integrity Number, RIN (Agilent Technologies) or the RNA Quality Score, RQI (BioRad). These scores vary between 0 (RNA totally degraded) and 10 (‘perfect’ RNA). A value of seven has been suggested as a limit between ‘good’ and ‘bad’ quality RNA extracted from bacterial pure cultures (Jahn *et al*., 2008). However, RNA extracted from natural environments such as soil or sediment will likely have lower quality due to the more complex matrixes and often harsh extraction techniques routinely used, for example bead beating (Hurt *et al*., 2001), but this information is not widely reported in the literature. Nevertheless, as highlighted above, even if reported, a shortcoming for RIN/RQI algorithms is that they are primarily based on rRNA (16S/23S ratio) which may degrade differently from mRNAs; the relevance of such indexes for gene expression analysis is therefore unknown.

In Eukaryotic gene expression studies, an alternative index often used to evaluate mRNA degradation is the 3′‐5′ ratio (Die *et al*., 2011). This technique is based on the observation that Eukaryotic mRNAs generally degrade from the 5′ to the 3′ end, with the 3′ poly(A) tail acting as a protective agent. As a result, Reverse Transcriptase‐PCR (RT‐PCR) targeting the 5′ end of the transcript is less likely to produce amplicons than those targeting the 3′ end. A high 3′:5′ ratio (low 5′ copy number) is therefore an indication of mRNA degradation. This technique cannot be applied to prokaryotic mRNAs as they generally do not possess poly(A) tails, and when they do, the tail enhances mRNA degradation (Dreyfus and Régnier, 2002). Recently, a new approach called differential amplicon (Δamp) has been developed (Björkman *et al*., 2016). This technique is based on the differential amplification of RT‐PCR amplicons of different lengths from the same mRNA target as a new means to determine RNA integrity (see also Karlsson *et al*., 2016). Here, it was observed that the copy number of long RT‐Q‐PCR targets correlated with mRNA degradation whereas short targets were more stable. Since this approach does not rely on the presence of the poly(A) tail, it could theoretically be adapted to prokaryotic mRNA. Although degradation of longer transcripts, faster, has not been directly observed previously in prokaryotes, Reck and colleagues(2015) showed a similar response of an exogenous green‐fluorescent‐protein mRNA (GFP), spiked into stool RNA, to montior its intergery when subjected to different storage conditions. They showed that the copy number of the spiked exogenous GFP correlated well with RNA integrity when targeting long amplicon (≥500 bp), whereas the short amplicon (≤100 bp) remained constant, even in highly degraded RNA preparations. This indicated that, as was observed by Björkman and colleagues, longer mRNA targets reflect degradation better. As such, the difference in RT‐Q‐PCR performance, reflected by the difference in cycle threshold (Ct) between a short and a long amplicon from the same cDNA target could be used as an index to reflect mRNA integrity.

Here, we propose to exploit the differential amplicon approach, initially developed by Björkman and colleagues, to develop a ratio of long to short amplicons of Bacterial mRNA transcripts using universal primers targeting conserved regions of the ubiquitous bacterial glutamine synthetase A transcript (*glnA*) as an indicator of overall mRNA integrity. Glutamine synthetase is a ubiquitous gene, found in Bacteria and Archaea (Kumada *et al*., 1993; Brown *et al*., 1994), with a role in assimilating inorganic nitrogen (ammonia) into amino acids (Reitzer, 2003). The *glnA* transcript has been used previously in RT‐(Q)‐PCR approaches to evaluate RNA extraction yield from soils (Sessitsch *et al*., 2002; Costa *et al*., 2004; Sharma *et al*., 2012). However, as the expression of *glnA* is regulated by ammonia concentration (Atkinson *et al*., 2002; Hua *et al*., 2004; Leigh and Dodsworth, 2007), the copy number of this transcript can vary making comparison between samples difficult. Our approach overcomes this difficulty by calculating the ratio of long to short *glnA* transcripts. We designate this the Ratio Amplicon (*R*_amp_), and propose it as an indicator of mRNA integrity, independent of absolute gene expression.

Specifically, this study aims to design and test the Ratio Amplicon (*R*_amp_) approach to evaluate bacterial mRNA integrity extracted from marine surface mud samples (0 to 2 cm; Rusheen Bay, Ireland) using a phenol‐chloroform/bead‐beating co‐extraction method. Furthermore, we aim to compare and evaluate this approach against the conventional ribosomal based RNA Integrity Number, RIN. Comparison between the two approaches was conducted by monitoring how well both indexes reflected experimental RNA degradation (UV, heat, *RNase*, freeze/thaw and long‐term storage). The impact of RNA degradation and the ability of the two indexes to predict ribosomal and mRNA integrity was evaluated via quantification of two commonly surveyed bacterial transcripts, the highly abundant ribosomal 16S rRNA and mRNA from the less abundant bacterial ammonia monooxygenase (*amoA*). Finally, the effect of RNA degradation on transcript community structure was evaluated by amplicon sequencing of the cDNA obtained from sequentially degraded samples.

We hypothesized that (i) the *R*_amp_ would be a better predictor of mRNA integrity than the RIN and (ii) RNA degradation would adversely affect both transcript quantification and community composition.

## Results

### *Design and optimization of* glnA *primers*

**Figure 1:**
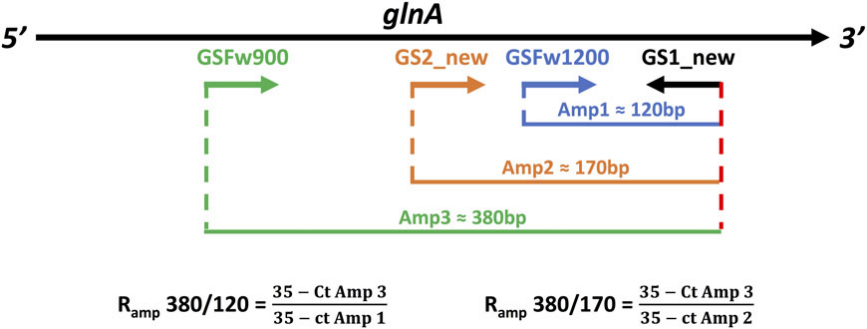
Schematic representation of primer binding sites along the Bacterial *glnA* gene. Primers are represented by arrows pointing to the right (forward primers) or to the left (reverse primer). The amplicons (Amp) generated by the different primer combinations are represented as coloured lines. The formulas used to calculate the two *R*_amp_ indexes are detailed under the figure.

Three new forward *glnA* primers (GSFw1200, GSFw900 and GSFw800) were designed to target a conserved region in groups 3, 4, 5, 7 and 8 of the *glnA* alignment (Table 1) at ≈120 bp, ≈380 bp and ≈500 bp, respectively, in front (closer to the 5′ end of the gene) of an updated reverse primer from Hurt and colleagues (2001) named, GS1_new primer. This resulted in three amplicon sizes to derive a ratio amplicon (*R*_amp_) from (Fig. 1). The newly designed primers (Table 1) were optimized for PCR and RT‐PCR resulting in amplicons of the expected size for all primer pairs. Assays were subsequently optimized for SYBR Green Q‐PCR. All primers except for GSFw800, producing the 500 bp amplicon were successfully optimized with diagnostic single peak melt curves. As such we proceeded with two *R*_amp_ ratio primer sets the *R*_amp_ 380/120 and the *R*_amp_ 380/170.

**Table 1:** List of primers used in this study.

Primer	Sequence (5′ ➔ 3′)	Orientation	Target	Experiment	Reference
GS1_new	GCTTGAGGATGCCGCCGATGTA	Reverse	Bacterial *glnA*, all amplicons	Q‐PCR and sequencing	This study, modified from Hurt *et al.,* 2001
GSFw1200	GGTTCGGGCATGCACGTGCA	Forward	Bacterial *glnA*, amplicon 1 (120 bp)	Q‐PCR	This study
GS2_new	AAGACCGCGACCTTNATGCC	Forward	Bacterial *glnA*, amplicon 2 (170 bp)	Q‐PCR	This study, modified from Hurt *et al.,* 2001
GSFw900	GTCAARGGCGGYTAYTTCCC	Forward	Bacterial *glnA*, amplicon 3 (380 bp)	Q‐PCR and sequencing	This study
GSFw800	GAAGCCGAGTTCTTCSTCTTCGA	Forward	Bacterial *glnA*, amplicon 4 (540 bp)	PCR	This study
BacamoA‐1F	GGGGHTTYTACTGGTGGT	Forward	Bacterial *amoA* gene (435 bp)	Q‐PCR and sequencing	(Hornek *et al*., 2006)
BacamoA‐2R	CCCCTCBGSAAAVCCTTCTTC	Reverse			
1369F	CGGTGAATACGTTCYCGG	Forward	Bacterial 16S rRNA gene (123 bp)	Q‐PCR	(Suzuki *et al*., 2000)
1492R	GGWTACCTTGTTACGACTT	Reverse			
1389P	CTTGTACACACCGCCCGTC	Probe			
F63	CAGGCCTAACACATGGCAAGTC	Forward	Bacterial 16S rRNA V1➔V3 (455 bp)	PCR	(Marchesi *et al*., 1998)
518R	ATTACCGCGGCTGCTGG	Reverse			(Lee *et al*., 2010)
515F	GTG YCA GCM GCC GCG GTA A	Forward	Bacterial 16S rRNA V4 (291 bp)	Sequencing	(Parada *et al*., 2016)
806R	GGA CTA CNV GGG TWT CTA AT	Reverse			(Caporaso *et al*., 2010)

### *Heat degradation*

**Figure 2:**
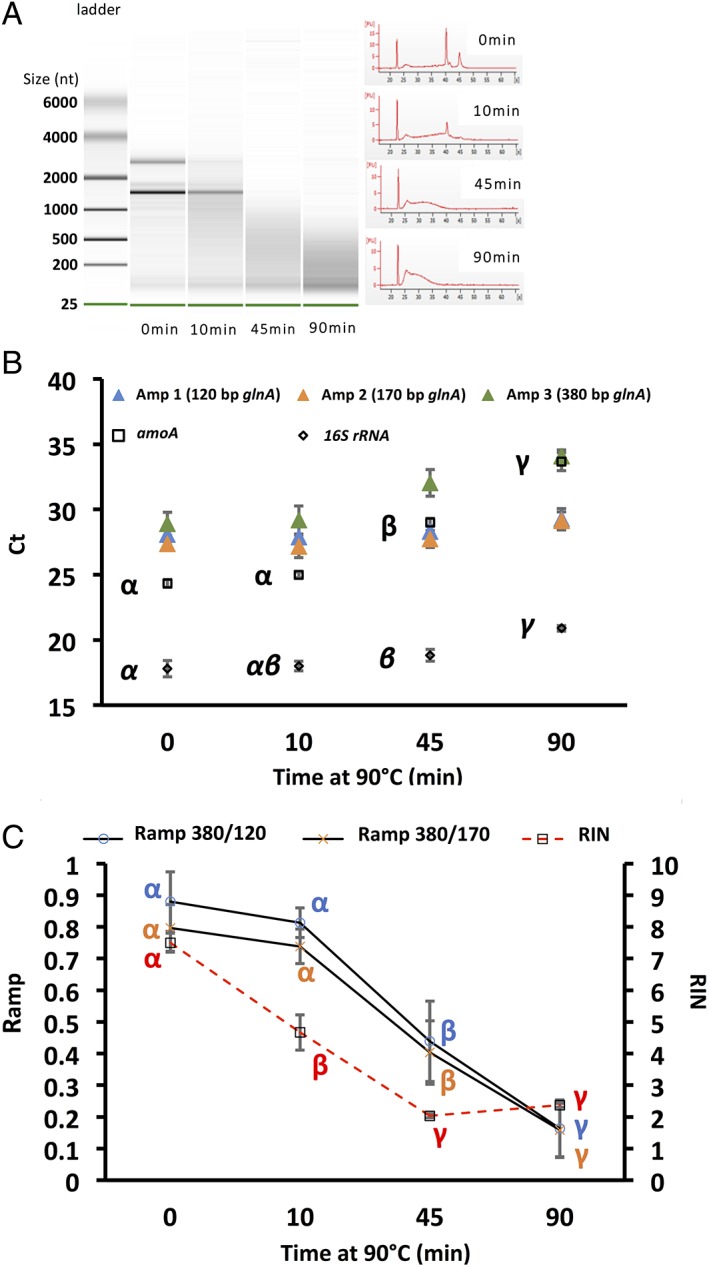
Effect of heat degradation on RNA integrity measured via the RIN (A), with RT‐Q‐PCR (B) and RIN versus *R*_amp_ (C). For RIN, RNA integrity visualized in virtual gels (A; left) and electropherogram (A; right) are displayed against incubation period at 90°C. RNA ladder shows size in nucleotides (nt). B. Effect of degradation on transcript quantification; Amp 1–3: average Ct (*n* = 3) of one of the three possible *glnA* amplicons; *amoA*: average *amoA* Ct (*n* = 3) of the Bacterial *amoA* transcript; *16S rRNA*: average *16S rRNA* Ct (*n* = 3) of the bacterial *16S rRNA* transcript. Effect of RNA degradation on *R*_amp_ index is presented in figure C; for comparison, RIN values were also plotted. Greek Letters indicate the result of TukeyHSD tests (points with different letters had values significantly different from each other using 0.05 as threshold for the *p* value).

Incubation of RNA at 90°C had a strong and rapid impact on its integrity with a drop in the RIN from 7.5 to 4.7 after 10 min. At this point, the band corresponding to 23S rRNA had almost completely disappeared. Further exposure resulted in more pronounced degradation with accumulation of short RNA fragments and a RIN around 2 for both 45 min and 90 min exposure (Fig. 2A and C). One‐way ANOVA revealed significant difference between all time‐points, except 45 and 90 min. A low and non‐significant decrease in both *R*_amp_ indexes was observed (−0.07 for 380/120 and − 0.11 for 380/170) between 0 and 10 min (Fig. 2C). This would tend to indicate that the *R*_amp_ was less sensitive than the RIN for monitoring RNA degradation by heat. However, interestingly the increase in Ct was also not significant for both *amoA* and *16S rRNA* between 0 and 10 min (Fig. 2B), showing that the *R*_amp_ reflected the outcome of the RT‐Q‐PCR assays better than the RIN. Further exposure to heat induced a more pronounced decrease in both *R*_amp_ (≈ −0.4 for 380/120 and ≈ −0.3 for 380/170) at 45 min compared with 0 min. Both *R*_amp_ indexes reached values around 0.15 at 90 min, which mapped well the behaviour of *amoA*, with a sharp increase in the Ct for this transcript between 10 and 45 min (≈4cts) and between 45 and 90 min (another ≈4cts). The *16S rRNA* transcript was also affected but to a smaller extent (increase in Ct of only ≈3ct between 0 and 90 min). Yet, in this case too, the increase was quite low between 0 and 10 min and sharper between 10 and 45 min and 45 and 90 min.

### *UV degradation*

**Figure 3:**
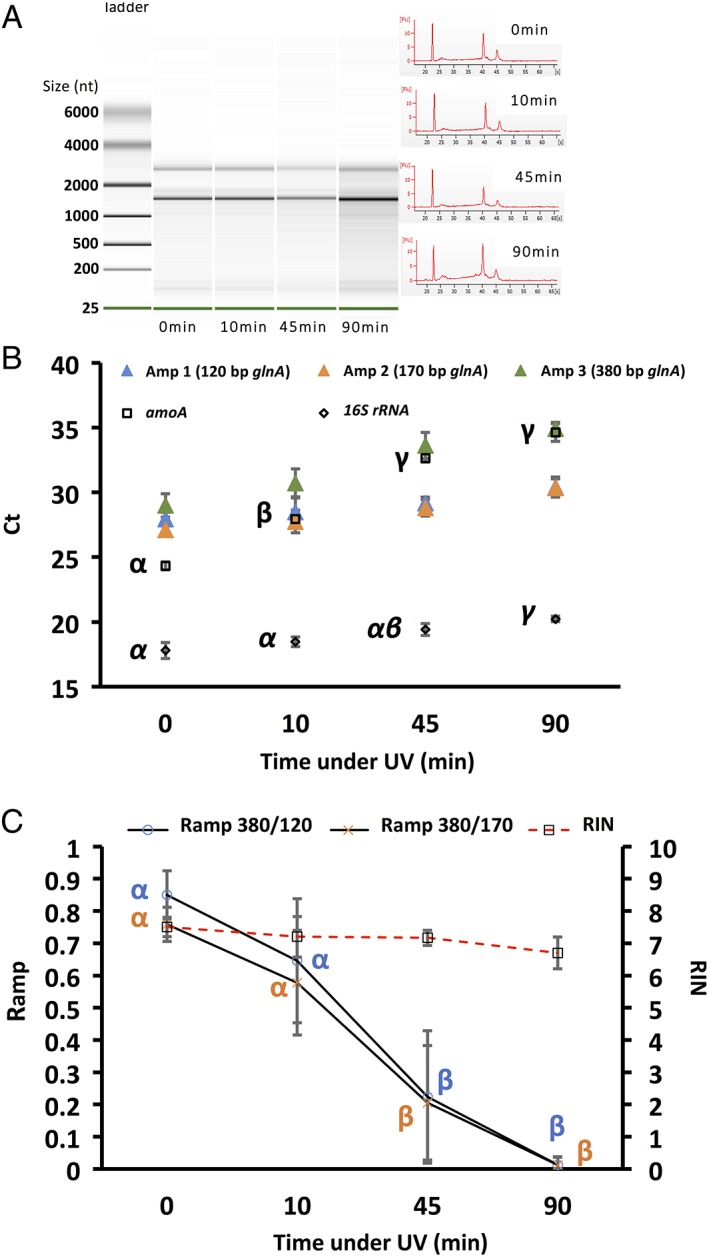
Effect of UV degradation on RNA integrity measured via the RIN (A), with RT‐Q‐PCR (B) and RIN versus *R*_amp_ (C). For RIN, RNA integrity visualized in virtual gels (A; left) and electropherogram (A; right) are displayed against incubation period under UV. RNA ladder shows size in nucleotides (nt). B. Effect of degradation on transcript quantification; Amp 1–3: average Ct (*n* = 3) of one of the three possible *glnA* amplicons; *amoA*: average *amoA* Ct (*n* = 3) of the Bacterial *amoA* transcript; *16S rRNA*: average *16S rRNA* Ct (*n* = 3) of the bacterial *16S rRNA* transcript. Effect of RNA degradation on *R*_amp_ index is presented in figure C; for comparison, RIN values were also plotted. Greek Letters indicate the result of TukeyHSD tests (points with different letters had values significantly different from each other using 0.05 as threshold for the *p* value).

The RIN was almost insensitive to UV radiation with an overall decrease of ≈1 at 90 min compared with 0 min (Fig. 3A and C). In contrast, UV radiation had a more pronounced effect on transcript quantification than heat as reflected by a quasi linear increase in Ct of the *amoA* transcript between 0 and 45 min (Fig. 3B). Unlike heat exposure, 10 min under UV induced strong and significant increase in *amoA* Ct values (≈4cts). At 45 min, the Ct had increased by ≈9 compared with the starting point. After 90 min, the Ct of the *amoA* transcript almost reached 35, close to the detection limit. The Ct for *16S rRNA* transcript increased steadily from 18 at 0 min to 20 at 90 min, showing that this assay/transcript was less sensitive to UV degradation. The behaviour of the *R*_amp_, again, mapped well onto *amoA* behaviour with a decrease of ≈0.2 after 10 min exposure for both indexes (although this was not significant) (Fig. 3C). A net decrease was observed at 45 min (≈ −0.6 compared with 0 min) and at 90 min both *R*_amp_ almost reached 0 since the Ct of the amplicon 3 *glnA* (380 bp) was very close to 35.

### *Degradation by* RNase I

**Figure 4:**
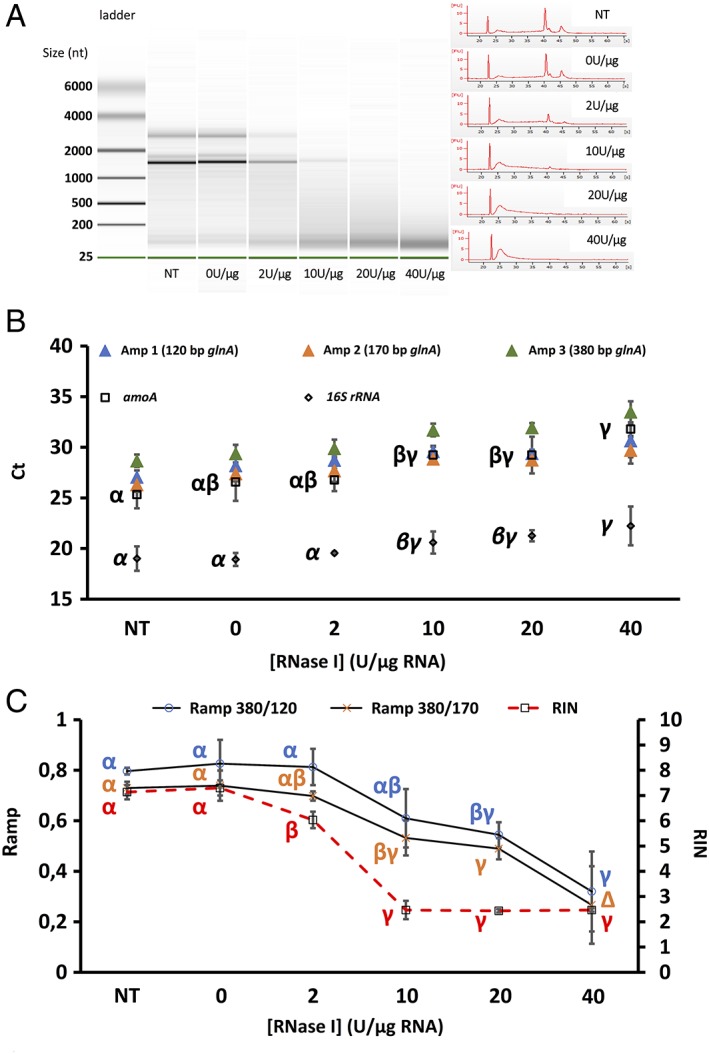
Effect of *RNase I* degradation on RNA integrity measured via the RIN (A), with RT‐Q‐PCR (B) and RIN versus *R*_amp_ (C). For RIN, RNA integrity visualized in virtual gels (A; left) and electropherogram (A; right) are displayed against incubation period with *RNase I*. RNA ladder shows size in nucleotides (nt). B. Effect of degradation on transcript quantification; Amp 1–3: average Ct (*n* = 3) of one of the three possible *glnA* amplicons; *amoA*: average *amoA* Ct (*n* = 3) of the Bacterial *amoA* transcript; *16S rRNA*: average *16S rRNA* Ct (*n* = 3) of the bacterial *16S rRNA* transcript. Effect of RNA degradation on *R*_amp_ index is presented in figure C; for comparison, RIN values were also plotted. Greek letters indicate the result of TukeyHSD tests (points with different letters had values significantly different from each other using 0.05 as threshold for the *p* value).

The RIN showed a rapid response to *RNase I* degradation with a decrease from 7.1 to 6 between 0 and 2 U μg^−1^ (Fig. 4A and C.) as seen on virtual gels and electropherograms with an almost complete disappearance of the 23S rRNA. When using 10 U μg^−1^ and higher concentrations, the RIN decreased and remained stable at approximately 2.5 indicating advanced/almost complete degradation of the RNA. Complete destruction of both rRNA and an accumulation of small size RNA molecules on the electropherogram can be observed (Fig. 4A). In contrast, enzymatic degradation by *RNase I* had a relatively small effect on the Ct of the *amoA* transcript at low concentration (only 0.2 Ct increase between 0 and 2 U μg^−1^ treatments) (Fig. 4B). Ct values for *amoA* increased with greater degradation of the parent RNA (3 Cts difference at 10 and 20 U μg^−1^ and 5 Cts at 40 U μg^−1^ compared with 0 U μg^−1^ control). Of note, *amoA* transcripts were still quantified from the degraded 40 U μg^−1^ treatment with a mean Ct of 31.8. *RNase I* seemed to be the most effective treatment for the destruction of rRNA. Indeed, an increase of ≈ 3.2 Cts for the *16S rRNA* transcript was observed between 0 and 40 U μg^−1^ treatments whereas an increase of only 2.2 Cts was observed between 0 and 90 min for both physical degradation techniques (heat and UV). *R*_amp_ indexes were only slightly affected by 2 U *RNase I* μg^−1^ (decrease of ≈0.015 for 380/120 and ≈0.03 for 380/170) (Fig. 4C). The decrease was more pronounced for both *R*_amp_ at higher concentrations of *RNase I* (≈0.25 decrease at 20 U μg^−1^ compared with 0 U control). Even at concentrations as high as 40 U μg^−1^, the *R*_amp_ indexes only reached 0.3. This indicated that at the high nuclease concentrations, even the small amplicons (120 and 170 bp) were starting to degrade. In this experiment, the *R*_amp_ 380/170 seemed to be more sensitive than the *R*_amp_ 380/120 in mapping RNA degradation, with significant differences between 0 and 10 U μg^−1^ treatments whereas *R*_amp_ 380/120 values only became significantly different from 0 U control from 20 U μg^−1^. Again, as observed in the other degradation experiments, the behaviour of the *amoA* Ct was better reflected by changes in *R*_amp_, especially *R*_amp_ 380/170, rather than by changes in the RIN.

### *Effect of freeze/thaw cycles and storage*

The effect of repeated cycles of freeze thaw on RNA is still poorly understood (and rarely studied) as conflicting results are reported, yet this is a common cause for concern when working with RNA. In our experiments, repeated freeze/thaw cycle (up to 10) did not induce any noticeable effects on RNA integrity, whether monitored via RIN or *R*_amp_ (Supporting Information Additional file 4, Fig. S1). The effect of long‐term storage was also investigated, by monitoring the RIN and *R*_amp_ of the same RNA after 0, 1 and 4 months stored at −80°C. No statistically significant change in RIN or *R*_amp_ was observed (Supporting Information Additional file 4, Fig. S2).

### *Comparison between* R*_amp_ and RIN*

**Figure 5:**
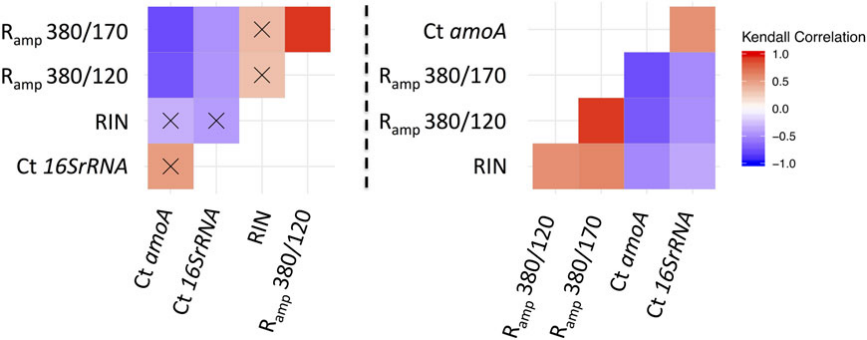
Kendall correlations between integrity indexes and Cts of the two reference gene used in this study. The correlations coefficients were calculated using all data generated from UV, heat and *RNase I* degradation experiments (left) and from the heat and RNase I only (right). The colour of squares represents the value of the correlation coefficients as explained on the colour scale. Black crosses indicate absence of significant correlation (threshold: *p* value > 0.05).

Data generated from all of the degradation experiments undertaken (UV, heat and *RNase I*) was compiled to determine which of the two integrity indexes (RIN vs. *R*_amp_) reflected the degradation status of the *amoA* and 16S rRNA transcripts more closely as determined by RT‐Q‐PCR. This was done by calculating Kendall correlations between either the *R*_amp_ or the RIN and the Cts of the two gene transcript targets (Fig. 5). When considering all three degradation experiments, that is UV, heat and *RNase I*, the RIN was not significantly correlated with 16S rRNA nor *amoA* Ct values (*p* value > 0.05). In contrast, both *R*_amp_ ratios resulted in a significant correlation with both *amoA* and 16S rRNA transcripts (Fig. 5). However, as the RIN was almost insensitive to UV, with a decrease of only about ≈1 after 90 min exposure (Fig. 3), Kendall correlations were repeated without the inclusion of the UV data set. In this case, both the RIN and the *R*_amp_ were significantly correlated with 16S rRNA and *amoA* transcript abundances within the degraded RNA samples (Fig. 5). In fact, the RIN was better correlated with *amoA* than 16S rRNA Cts. Nevertheless, both *R*_amp_ ratios were more highly correlated with *amoA* Cts than the RIN. Furthermore, the *R*_amp_ ratios were more highly correlated with the 16S rRNA than the RIN. Taken together, these two observations confirm that the *R*_amp_ indexes better reflected RT‐Q‐PCR changes induced by RNA degradation than the RIN.

### *Effect of RNA degradation on transcript community composition*

**Figure 6:**
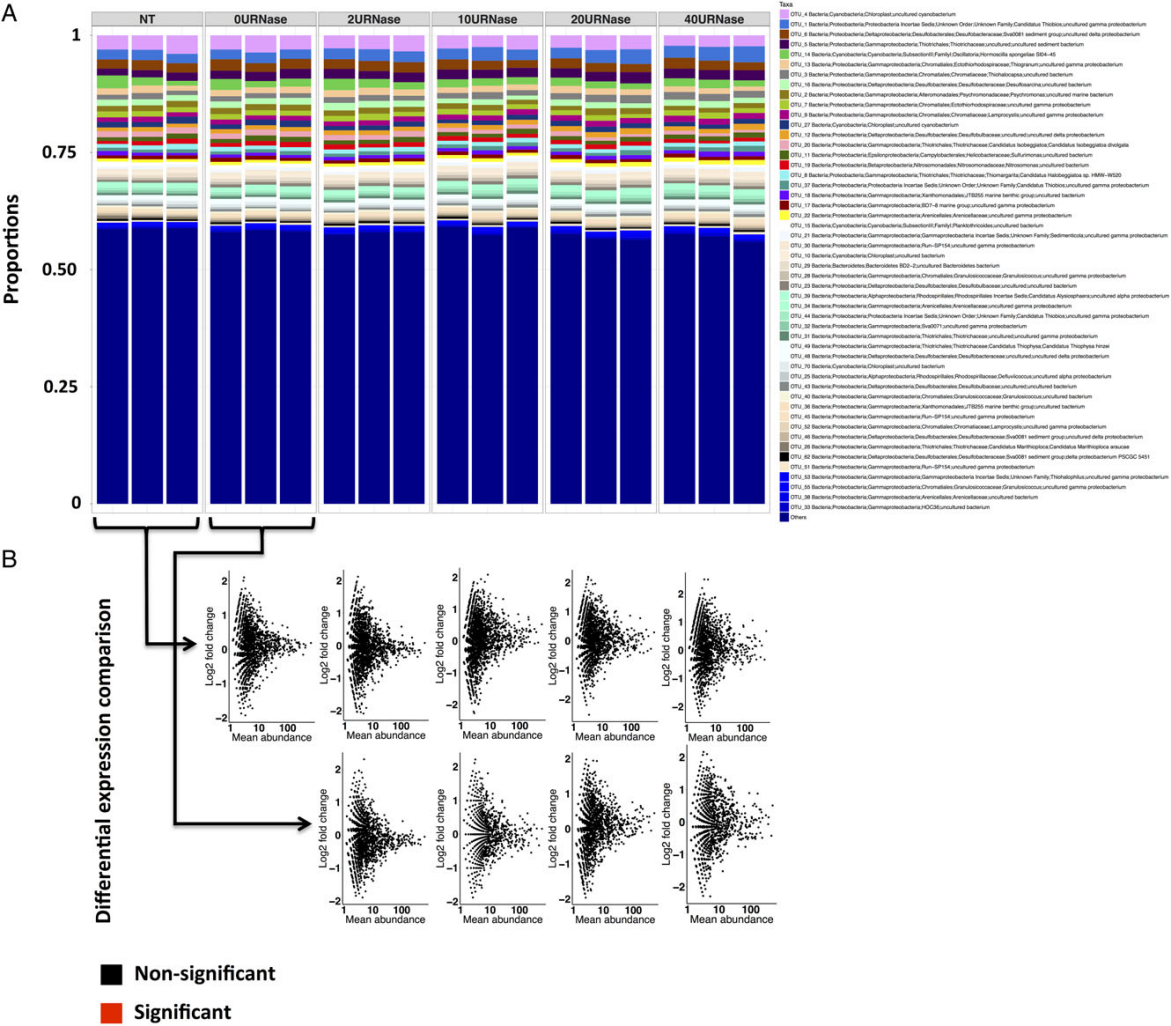
Effect of *RNase I* treatment on 16S rRNA transcript composition. Bar charts (A) represent changes in community composition of the 50 most abundant taxa. Scatterplots (B) represent log2 changes of individual taxa along the degradation gradient relative to control experiments (no treatment control (NT) or buffer only control (0U*RNase I* μl^−1^)) as indicated by black arrows. Taxa with a significant difference (*p* value < 0.05) in expression greater than or equal to a twofold change (positively or negatively) relative to controls are indicated in red.

**Figure 7:**
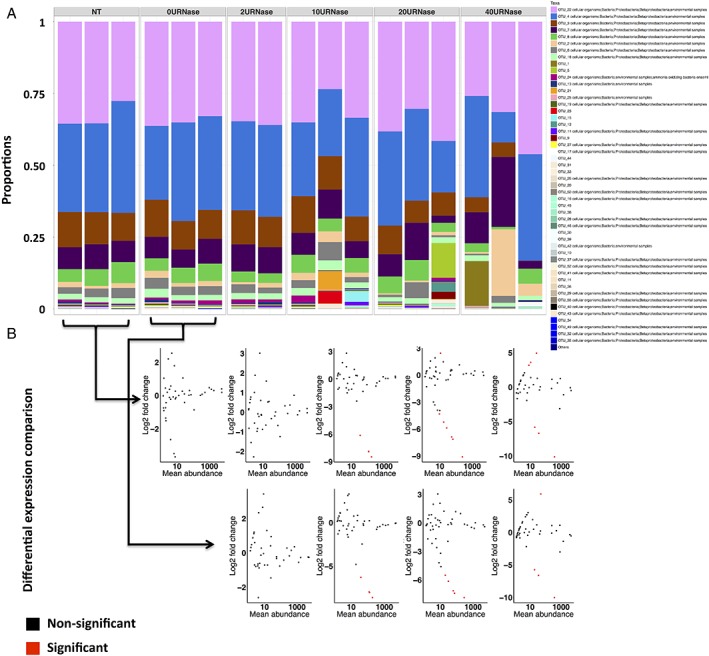
Effect of *RNase I* treatment on *amoA* transcript composition. Bar charts (A) represent changes in community composition of the 50 most abundant taxa. Scatterplots (B) represent log2 changes of individual taxa along the degradation gradient relative to control experiments (no treatment control (NT) or buffer only control (0U*RNase I* μl^−1^)) as indicated by black arrows. Taxa with a significant difference (*p* value < 0.05) in expression greater than or equal to a twofold change (positively or negatively) relative to controls are indicated in red.

**Figure 8:**
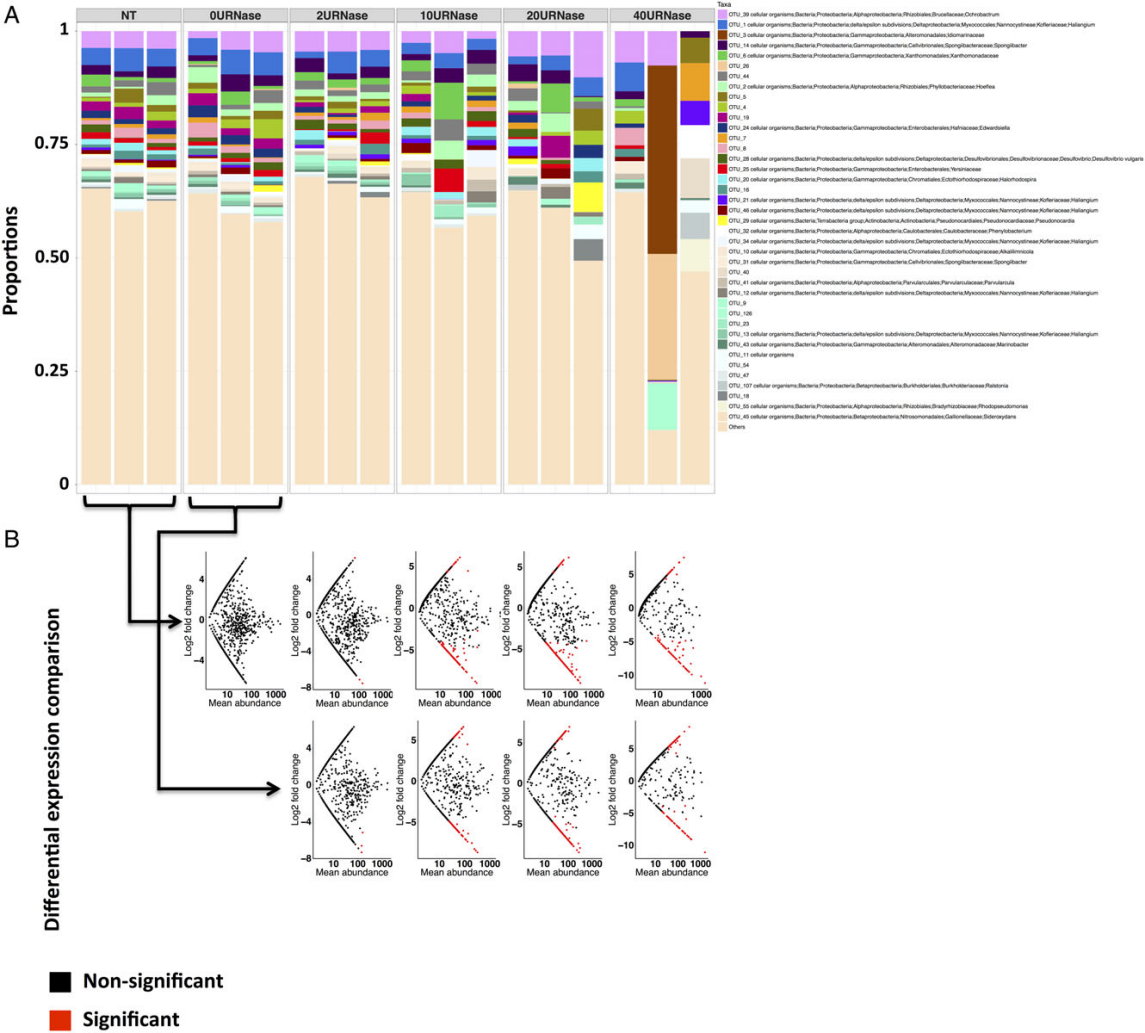
Effect of *RNase I* treatment on *glnA* transcript composition. Bar charts (A) represent changes in community composition of the 50 most abundant taxa. Scatterplots (B) represent log2 changes of individual taxa along the degradation gradient relative to control experiments (no treatment control (NT) or buffer only control (0U*RNase I* μl^−1^)) as indicated by black arrows. Taxa with a significant difference (*p* value < 0.05) in expression greater than or equal to a twofold change (positively or negatively) relative to controls are indicated in red.

RNA degradation impacted upon *amoA, glnA* and *16S rRNA* gene quantification, as demonstrated previously. However, whether all members of the community were affected equally was still to be determined. To answer this question, cDNA amplicons of the Bacterial 16S rRNA, *amoA* and *glnA* transcripts underwent Illumina MISeq amplicon sequencing from all degradation points of the *RNase I* experiment representing RNA with RIN values from 7.5 to 2.4 and *R*_amp_ values from ≈0.8 to ≈0.3 and from ≈0.7 to ≈0.3 for *R*_amp_ 380/170 and *R*_amp_ 380/120 respectively. The effect of *RNase I* treatment on community evenness was tested using PERMANOVA. Results are presented in Figs 6, 7 and 8. Interestingly, the community structure of the three transcripts studied responded differently.

Strikingly, *RNase I* treatment had little effect on *16S rRNA* transcript community evenness (Fig. 6A). Indeed, for individual OTU, none of the members of the community were significantly differentially represented (*p* value log_2_ difference > 0.05) within highly degraded samples in comparison to controls (Fig. 6B). For individual OTU, at least 90% had their relative expression change over the degradation experiment fall within the [−log2(1.5); log2(1.5)] interval, even when comparing controls to the completely degraded 40 U *RNase I* sample (Fig. 6B). This indicates that *16S rRNA* OTU transcript community was responding evenly to degradation, with each member having the same chance to be affected regardless of its abundance or sequence.

For bacterial *amoA* transcript community, there was no change in the overall composition with increasing degradation as reflected by the non‐significant PERMANOVA (*p* value > 0.05) (Fig. 7). However, with increasing degradation, there was an increasing difference in the community evenness among replicates. Furthermore, unlike 16S rRNA transcripts, when examining individual *amoA* OTUs it was evident that in the degraded samples some OTUs were differentially represented at a significant level compared with controls (Fig. 7B). In fact, some OTUs in the highly degraded samples (10, 20 and 40 U *RNase I*) had a fold change difference of up to two orders of magnitude compared with the controls and in most cases, resulting in their over representation in degraded samples (see Supporting Information Additional file 3). Moreover, in the more highly degraded treatments (10, 20 and 40 U *RNase I*), up to 44% of *amoA* OTUs had their relative expression outside the [−log2(1.5); log2(1.5)] interval, compared with the starting RNA (Fig. 7 B). So, while there was not an overall significant difference in *amoA* community structure with increasing RNA degradation, there were changes in the relative expression of individual OTU. The lack of overall statistical significance in community structure may in fact be explained by the overall lower numbers of *amoA* OTUs for comparison and the increasing difference among replicates in the degraded samples.

The effect of *RNase I* treatment was much more pronounced for *glnA* transcripts, than for *amoA,* and a significant change in community composition with increasing degradation was observed (*p* value < 0.05 for PERMANOVA with both Bray‐Curtis and Unifrac distances) (Fig. 8A and B). As seen with *amoA*, the difference in community composition between replicates also increased with increasing *RNase I* treatment. Moreover, this effect was also observed at individual OTU level with a large fraction of the individual OTU showing different expression levels in treated samples compared with controls (Fig. 8B). As seen for *amoA*, some *glnA* OTUs were highly over represented in degraded samples by 2 to 3 orders of magnitudes (Supporting Information Additional file 3), e.g. when comparing the untreated samples (NT) to the 40U*RNase* samples, 0.28% (3 sequences) were over represented by two orders of magnitude. When comparing the samples treated with buffer only to the 40U*RNase* samples, 2.43% (19 sequences) were over represented by two orders of magnitude and 0.13% (1 sequence) by three orders of magnitude.

## Discussion

Here, we successfully designed and tested the Ratio Amplicon, *R*_amp,_ index. The concept is that as RNA degrades, longer strands are preferentially affected and the abundance of the longer amplicon relative to the shorter amplicon will decrease with increasing RNA degradation (Björkman *et al*., 2016). Using experimentally degraded environmental RNA, we have shown that the newly developed *R*_amp_ index was a better predictor of the Ct of the target mRNA transcript used in this study, *amoA,* than the ribosome‐based RIN approach. In fact, when data from the three degradation experiments carried out was considered together only the *R*_amp_ statistically correlated with *amoA* Cts. As the RIN failed to detect UV degradation, we removed this data from the correlation calculation to determine if this data set was biasing the results towards the *R*_amp_ approach. In this case, there was also a significant correlation between the RIN and *amoA* Ct (−0.51). However, the *R*_amp_ index still reflected the fate of the mRNA better than the RIN (−0.72 and − 0.77 for *R*_amp_ 380/120 and *R*_amp_ 380/170 respectively).

Taking the different RNA degradation approaches used individually, the RIN and *R*_amp_ ratios responded differently. As noted above, the RIN did not change over a 90‐min exposure to UV. UV causes intramolecular cross‐linking of thymines but does not cause strand breaks (Kladwang *et al*., 2012) while the RIN monitors stand break. Similar results were obtained by Bjorkman *et al* (Björkman *et al*., 2016) who reported a lack of response for the RIN and the RQI when human RNA preparations were degraded by UV radiation, even after 120 min of exposure. As such RNA damage by UV cannot be detected by electrophoresis separation but is recorded by RT‐Q‐PCR *R*_amp_ index. Other RNA degradation processes that result in base destruction but not necessarily strand break include oxidative damage (Rhee *et al*., 1995) or chemically‐induced radical formation (Hawkins and Davies, 2002).

In contrast, the RIN was the most efficient method to detect heat degradation. There was a strong and significant decrease in this index after 10 min whereas the *R*_amp_ indexes only became significantly different from the controls after 45 min. Moreover, there was very little effect on the direct quantification of the transcripts by RT‐Q‐PCR with very little change in the Ct of either *amoA* or 16S rRNA in the first 10 min at 90°C. Initially, heat degradation caused a rapid decrease in the RIN. However, at this point the RT‐Q‐PCR targets were actually responding more slowly and were more closely mapped by the *R*_amp_ than the RIN. Björkman *et al*., 2016 showed a similar response of their differential amplicon, the ΔΔamp index, that did not change much between 2 and 10 min at 95°C whereas the RIN rapidly reduced from 7 to 2. Moreover, Gingrich *et al* (Gingrich *et al*., 2008) showed that transcripts could be quantified from RNA preparations incubated at 90°C for several hours. This relatively low impact of heat on RNA quantification may be due to modification of RNA secondary structures which could result in more efficient cDNA synthesis and mask the effect of the heat‐induced reduction of RNA integrity. More likely it is due to the small amplicon size of the targets that are unaffected by degradation. This essentially illustrates the difference in the methods used to monitor RNA degradation – the RIN detects strand break no matter where the fracture occurs along the transcript while the *R*_amp_ will only detect degradation if the break occurs between primer binding sites.

RNA degradation using the nuclease enzyme *RNase I* was monitored using both RIN and *R*_amp_. A similar behaviour could be observed here as in the heat degradation experiment with the RIN responding more quickly but loosing sensitivity when RNA was highly degraded whereas the *R*_amp_ responded slightly later but remained sensitive when RNA was extensively degraded. *RNase I* was the degradation method that had the strongest effect on the 16S rRNA Ct. *RNase I* activity is dependent on the concentration of the substrate. If rRNA and mRNA are considered as two distinct substrates, it can be expected that *RNase I* will have a greater impact on ribosomes as they constitute 80%–85% of total RNA. Furthermore, cDNA synthesis from mRNA would be enhanced in preparations where rRNA was depleted (Petrova *et al*., 2017). This dynamic may mask and change the effect of degradation over time, which would explain the relatively low increase in Ct for *amoA* at the beginning of the *RNase I* degradation experiment. Nevertheless, in this experiment and generally, for all degradation tests carried out, the behaviour of the *amoA* Ct was better predicted by the *R*_amp_, as reflected by the higher correlation coefficient between *R*_amp_ indexes and *amoA* Ct than the RIN (Fig. 5). As the *in vitro* half‐life of different transcripts is not well‐understood and has been shown to vary (Selinger *et al*., 2003; Belasco, 2010; Evguenieva‐Hackenberg and Klug, 2011) further work is required to test the correlation of the *R*_amp_ against a larger range of mRNAs. For ribosomal RNA, while the correlation between the *R*_amp_ index and 16S rRNA Ct was lower than for *amoA,* it still correlated better with RNA degradation than the RIN. This indicates that the outcome of 16S rRNA analysis was less affected by degradation than our mRNA targets. There are two factors that may contribute to this, the reported greater robustness of ribosomal RNA than mRNA and the shorter (~103 bp) 16S rRNA amplicon. That ribosomal RNAs behave the same as mRNA has never been proven. On the contrary, Sidova and colleagues (2015) showed that when natural *post mortem* degradation occurs, rRNA is more stable than mRNA. In this case, rRNA is a poor predictor of degradation of the mRNA fraction, as supported by this work. As mRNA is subjected to more rapid decay to adjust to the needs of the cell whereas rRNA are degraded only under certain stress conditions or when defective (Deutscher, 2006), then these intrinsic differences in stability properties may also affect degradation rates of the different class of RNA post‐extraction. Therefore, based on this work we can conclude that the *R*_amp_ was a better predictor of mRNA integrity than the RIN. However, as we and others (Björkman *et al*., 2016) have shown RNA responds differently to different types of degradation e.g. strand break versus intramolecular cross‐linking of thymines, and as the exact and likely multiple causes of post‐extraction degradation are unknown, we recommend that the RIN is used in conjunction with the *R*_amp_ to monitor RNA integrity.

### *Which* R*_amp_ to use?*

In theory, the greater the difference between the two amplicons the more sensitive the *R*_amp_ index. However, as the *R*_amp_ approach is based on RT‐Q‐PCR it is restricted by the presence of conserved sites for primer design, and the success and efficiency of the RT and qPCR reactions. We initially designed a 500 bp *glnA* PCR amplicon however, the Q‐PCR assay failed to produce a single diagnostic melt curve analysis. Of the remaining shorter *R*_amp_ sets, in practice, only one *R*_amp_ index is necessary, we recommend using the *R*_amp_ 380/170. The *R*_amp_ 380/170 always had a higher value than the *R*_amp_ 380/120 which would indicate that the number of 170 bp targets is higher than the 120 bp. Since both are amplified from the same target, this is not possible and the explanation for this observation is the lower efficiency of the 120 bp Q‐PCR compared with the 170 bp assay. In spite of this, both *R*_amp_ correlated similarly well overall with each degradation experiment, with *R*_amp_ 380/170 slightly more sensitive in the *RNase I* experiment.

### *Impact of experimental degradation of environmental RNA on ribosomal (16S rRNA) and mRNA (*amoA *and* glnA*) community diversity*

For complex environmental communities, the integrity of RNA is not only important to evaluate quantitative gene expression but is also of significance if it adversely affects the relative abundance of transcript diversity. To examine this, we assessed changes in the community structure of the 16S rRNA, *amoA* and *glnA* transcripts from all fractions of the *RNase I* sequentially degraded RNA.

The results were surprising with successful amplicon sequencing even from highly degraded samples. Nevertheless, the data did suggest a different response of 16S rRNA and mRNA transcripts to degradation, with 16S rRNA community structure unaffected over the range of degraded RNA samples. That is a statistically similar community was present in the control non‐degraded samples as in the totally destroyed 40 Units *RNase I* (with a mean RIN of 2.5 and *R*_amp_ of 0.32 and 0.27 for *R*_amp_ 380/120 and *R*_amp_ 38/170 respectively). This indicates that while total RNA was degraded, the small transcript fragments required for RT‐PCR and amplicon sequencing remained intact. In fact, so much so that no significant change in the relative abundance of individual OTU was observed.

On the other hand, RNA degradation had a greater influence on both *amoA* and *glnA* mRNA targets. While, again surprisingly, transcript amplicons were successfully detected from all degradation status samples, greater variability between degraded replicates was observed. This resulted in statistically different communities for *glnA* but not *amoA* when compared with the same non‐degraded control samples. However, the low number of *amoA* OTUs and increased variability between replicates contributed to the lower statistical power resulting in no statistical difference between treatments (Fig. 7). Furthermore, there were significant, sometimes up to two to three orders of magnitude change in the relative abundance of individual *glnA* and *amoA* OTUs in the degraded samples versus control samples. So, while we could successfully amplify mRNA transcripts from degraded environmental samples, we have shown that the relative composition of the community members was adversely affected by degradation and was not representative of the initial starting point. While further work is needed to determine the impact of degradation across the entire transcriptome to see if all mRNAs respond in a similar manner, it is clear from our mRNA amplicon sequencing that RNA degradation will alter the outcome of community analysis. It is therefore necessary to ensure the RNA integrity of the sample is known prior to interpretation of results. For this, our data indicates that a combination of approach targeting both ribosomal (the RIN) and mRNA (the *R*_amp_) is needed.

### *Best practice for environmental RNA*

The challenge when working with environmental samples will always be to retrieve RNA of a high enough quality and integrity. Here, we started with RNA extracted from marine sediments that had an average RIN of ≈7 and *R*_amp_ of ≈0.8. This is the best quality RNA we could produce with this bead‐beating co‐extraction method (Griffiths *et al*., 2000) and it already falls at the lower end of acceptable RIN for pure culture (Jahn *et al*., 2008). Therefore, methods to improve the initial quality of RNA extractions should also be a high priority, although this will be easier in some environments than others. Improvement of extraction methods is crucial as it can lead to important differences in the results. For example, Feike *et al* (Feike *et al*., 2012) showed that different sampling techniques influenced the relative abundance of transcripts retrieved from the suboxic zone of the Baltic Sea. Next, the integrity of the extracted RNA should be determined, and it should be ensured that the integrity value is similar among samples to be compared. Here, the *R*_amp_ approach should be a useful tool to complement current electrophoretic approaches, such as the RIN prior to extensive downstream analysis.

Another consideration raised by this work is in the very fact that the differential amplicon approach works. This shows that small cDNA amplicons can still be produced from highly degraded RNA samples whereas long amplicons tend to disappear quickly. When using RNA samples of poor quality, the comparison of expression levels between different targets might be irrelevant if the difference in length of the RT‐Q‐PCR targets between genes is large. In this case, it would be better to use only small amplicons, that are less sensitive to degradation (Antonov *et al*., 2005). An alternative, to deal with samples with different degradation status, potentially could be to normalize RT‐Q‐PCR data to RNA integrity. A RIN based algorithm has been proposed by Ho‐Pun‐Cheung *et al* (Ho‐Pun‐Cheung *et al*., 2009) to reduce RT‐Q‐PCR errors due to RNA degradation in cancer biopsies. In our case, however, *R*_amp_ indexes correlated better than the RIN with *amoA* and 16S rRNA Cts, making them better potential candidates as normalization metrics. Therefore, we tested a normalization coefficient based on the *R*_amp_ (Supporting Information Additional file 4, Fig. S4). As in Ho‐Pun‐Cheung *et al.,* 2009, we assumed a linear relationship between the integrity index and the changes in transcript Cts (i.e. change in Ct = α × change in *R*_amp_). This assumption facilitated the calculation of a regression coefficient α that was used to normalize Cts as explained in Supporting Information Additional file 4, Fig. S4. Although the use of such normalization reduced the errors attributable to RNA degradation (Supporting Information Additional file 4, Fig. S4), several limitations remain: (i) the linear relationship between changes in Cts and *R*_amp_ might not always be true depending on the transcript tested, (ii) the regression coefficient α depends on the degradation technique (Supporting Information Additional file 4, Table S1), (iii) the regression coefficient α depends on the transcript tested (Supporting Information Additional file 4, Table S1) and (iv) the regression coefficient α may depend on the environment from which RNA was extracted. Until more work is done to validate such normalization strategies, or to dramatically improve the quality of the RNA that can be extracted from environmental samples (Feike *et al*., 2012), we recommend using integrity indexes (differential amplicon and microfluidics based techniques) as initial quality checks of RNA and advise not to make absolute comparisons among samples with dissimilar integrity status.

## Conclusion

Assessing RNA quality is essential for obtaining meaningful transcriptomic results. The current approach to monitor RNA integrity include the RIN and RQI. This is a useful technique that is widely under‐used (or reported) in microbial transcriptomics studies, to give an overview of total RNA quality based on a ratio between the 23S and 16S ribosomes. Since most transcriptomics studies are interested in the metabolic function and therefore mRNA, it would be preferable to have an integrity index to target the mRNA. Furthermore, it is unknown if degradation of rRNA reflects mRNA degradation. We therefore developed and experimentally tested a new index, the *R*_amp_, the goal of which was to specifically target mRNA degradation and we showed that it performed better than the RIN at predicting the outcome of RT‐Q‐PCR of a functional gene (*amoA*). It was shown in this study that both quantitative (RT‐Q‐PCR) and qualitative (sequencing) results can be obtained, even from very degraded samples. Comparison of gene expression level between preparations with different degradation levels can therefore lead to false conclusions if integrity is not checked prior to analysis. Thus, we encourage microbial ecologists to report integrity indexes in order to improve reproducibility and facilitate comparison between transcriptomics studies. For this we propose that a *R*_amp_ ratio is used alongside the RIN.

## Experimental procedures

### *Sediment samples*

Surface mud samples (0 to 2 cm) were collected on 11 January 2017 from Rusheen Bay, Ireland (53.2589° N, 9.1203° W) (presence of *amoA* genes/transcripts previously established (Duff *et al*., 2017; Zhang *et al*., 2018) in sterile 50 ml Eppendorf tubes, flash frozen and stored at −80°C until subsequent use.

### *Design of new* glnA *primers*

To design new primers, bacterial *glnA* sequences were downloaded from the GeneBank database (Clark *et al*., 2016). Sequences related to environmental bacteria were subjected to BLAST search (Altschul *et al*., 1990) in order to gather additional sequences. In total, 84 sequences (Supporting Information Additional file 1) were aligned using MUSCLE (Edgar, 2004) and a phylogenetic neighbour joining tree was drawn in MEGA 7 (Kumar *et al*., 2016). Based on sequence similarity, eight groups could be distinguished (see Supporting Information Additional file 4, Fig. S3). Primer sequences from Hurt and colleagues (2001) were aligned in each individual group to determine coverage and new primers (Table 1) were designed based on conserved regions to target the same groups with varying length primers.

Primers were tested on DNA and cDNA using environmental DNA/RNA extractions and environmental cDNA, as template. *glnA* genes were amplified (BIOTAQ DNA polymerase kit; Bioline) in a 25 μl final volume composed of 2.5 μl BioTaq10x buffer, 18 μl water, 1.5 μl MgCl_2_ (50 mM), 0.5 μl of each primer (10 μM), 0.5 μl dNTPs (10 μM each), 0.5 μl *Taq* DNA polymerase and 1 μl of template. PCR conditions were as follow: 95°C 5 min, (94°C 30 s, 60°C 30 s, 72°C 30 s) × 30 and 72°C 5 min.

### *RNA preparation from sediment*

All surfaces and equipment were cleaned with 70% ethanol and *RNase* Zap (Ambion) before sample processing. All glassware and stirrers used for solutions preparation were baked at 180°C overnight to inactivate *RNases*. All plasticware was soaked overnight in *RNase* away (ThermoFisher Scientific) solution. Consumables used, including tubes and pipet tips were *RNase* free. All solutions were prepared using Diethylpyrocarbonate (DEPC) treated Milli‐Q water. A simultaneous DNA/RNA extraction method based on that of Griffiths and colleagues (2000) was used to recover nucleic acids from sediment. Briefly, 0.5 g of sediments were extracted from using bead beating lysing tubes (Matrix tube E; MP Biomedical) and homogeneized in 0.5 ml CTAB/phosphate buffer (composition for 120 ml: 2.58 g K_2_HPO_4_.3H_2_O; 0.10 g KH_2_PO_4_; 5.0 g CTAB; 2.05 g NaCl) plus 0.5 ml Phenol:Chloroform:Isoamyl alcohol (25:24:1 v:v:v). Lysis was carried out on a FastPrep system (MP Biomedical) (S: 6.0; 40s) followed by a centrifugation at 12 000*g* for 20 min (4°C). The top aqueous layer was transferred in a fresh 1.5 ml tube and mixed with 0.5 ml chloroform:isoamyl alcohol (24:1 v:v). The mixture was centrifuged at 16 000*g* for 5 min (4°C) and the top aqueous layer was transferred in a new 1.5 ml tube. Nucleic acids were precipitated by adding two volumes of a solution containing 30% poly(etlyleneglycol)_6000_ (PEG6000) and 1.6 M NaCl for 2 h on ice and recovered by centrifugation at 16 000*g* for 30 min (4°C). The pellet was washed with 1 ml ice‐cold 70% ethanol and centrifuged at 16 000*g* for 30 min (4°C). Ethanol wash was discarded and the pellet was air dried. Once the ethanol was completely evaporated, the pellet was re‐suspended in DEPC treated water. DNA/RNA preparations were stored at −80°C if not used immediately.

RNA was prepared from the DNA/RNA co‐extraction by DNase treating with Turbo DNase Kit (Ambion) using the extended protocol: half the recommended DNase volume is added to the sample and incubated for 30 min at 37°C, after which the second half of DNase is then added and the sample is re‐incubated at 37°C for 1 h. Success of the DNase treatment was checked by no PCR amplification of the V1–V3 Bacterial *16S rRNA* gene (Smith *et al*., 2006).

### *RNA degradation experiments*

#### *Physical degradation*

To obtain RNA with controlled degradation status, DNA free RNA preparations (≈8 μl) were aliquoted from an initial extraction in separate 0.2 ml *RNase* free tubes and incubated at 90°C or under a UV lamp for 0, 10, 45 or 90 min. To determine the potential effect of repeated freeze–thaw on RNA preparations, the same 15 μl DNA‐free RNA was exposed to cycles of freezing (at −80°C) and thawing (on ice) as follows – 0, 1, 3, 5, 7 and 10 freeze–thaw cycles. cDNA was then generated for each individual aliquot as described later. Aliquots of RNA were stored at −80°C for up to 4 months, at time 0, 1 month and 4 months samples were removed from the freezer to determine RNA integrity.

#### *Enzymatic degradation by RNase I*

For *RNAse I* degradation experiment, 40 μl aliquots of DNA‐free RNA was incubated at 37°C for 40 min in the presence of increasing concentrations of *RNase I* (Ambion): 0 (buffer only), 2, 10, 20 and 40 Units *RNase I* μg^−1^ RNA. The reaction was stopped by adding 10 μl β‐mercaptoethanol and RNA was recovered by ethanol precipitation: 5 μl of 7.5 M ammonium acetate and 137.5 μl 100% ethanol was added and the mixture was precipitated overnight at −20°C. RNA was pelleted by centrifugation 16 000*g* for 40 min at 4°C and the pellet was washed with 480 μl ice cold 70% ethanol and pelleted by centrifugation at 16 000*g* for 30 min at 4°C. The pellet was air dried and re‐suspended in 40 μl of DEPC‐treated water. An aliquot of RNA that did not undergo ethanol precipitation was also included for comparison (designated NT: ‘Not Treated’).

### *Reverse transcriptase reaction*

DNA‐free RNA was used for *glnA* cDNA synthesis using Superscript III kit (Invitrogen) and gene specific priming. The initial RT mixture containing 3 μl water, 1 μl reverse primer GS1_new (10 μM), 1 μl dNTP's (10 mM each) and 5 μl template was incubated at 65°C for 5 min and quickly transferred on ice for 1 min. A second mix composed of 4 μl 5X first‐strand buffer, 1 μl 0.1 mM dithiothreitol (DTT) and 1 μl SuperScript III (200 units μl^−1^) was added and the resulting mixture was incubated at 55°C for 50 min and then at 72°C for 15 min. The primers and PCR conditions for the amplification of *glnA* targets from cDNA were similar to those used for DNA.

For *16S rRNA* and *amoA* genes, Superscript III kit (Invitrogen) and random hexamers priming was used. The initial RT mixture containing 3 μl water, 1 μl random hexamer (50 μM), 1 μl dNTP's (10 mM each) and 5 μl template was incubated at 65°C for 5 min and quickly transferred to ice for 1 min. A second mix composed of 4 μl 5X first‐strand buffer, 1 μl 0.1 mM dithiothreitol (DTT) and 1 μl SuperScript III (200 units μl^−1^) and 1 μl *RNase* inhibitor (40 U μl^−1^) was added and the resulting mixture was incubated at 25°C for 5 min, 55°C for 50 min and then at 72°C for 15 min.

### *RNA integrity evaluation*

#### *RNA integrity number*

RINs were determined at all degradation points, using the automated 2100 Bioanalyser platform (Agilent Technologies) with the Prokaryote total RNA Nano chip, following the manufacturer's instructions.

#### *glnA Q‐PCR and ratio amp (*R*_amp_) calculation*

*glnA* cDNA underwent Q‐PCR, to amplify varying length amplicon fragments with primer combination as detailed in Table 1. Three *glnA* amplicons were produced (Fig. 1), a 120 bp amplicon (amplicon 1) generated using the primer pair GS1_new/GSFw1200, a 170 bp amplicon (amplicon 2) generated using the primer pair GS1_new/GS2_new and a 380 bp amplicon (amplicon 3) generated using the primer pair GS1_new/GSFw900. Q‐PCR reaction (10 μl) was composed of 5 μl EVAGreen Supermixes (SsoFast; Bio‐Rad), 0.3 μl of each primers (10 μM) and 1 μl of cDNA template (1/10 diluted). The Q‐PCR condition was as follows: 95°C‐30s, (95°C‐10s; 65°C‐10 s) × 35 cycles; plate read at 65°C. Melt curve analysis was performed from 65°C to 95°C with 0.5°C increment every 5 s.

The Ct value of each assay was recorded and the differential amplicon ratios (*R*_amp_) were calculated for each degradation point as follows:

$$R_{\mathrm{amp}}=\frac{35-\mathrm{Ct}\left(\text{long amplicon}\right)}{35-\mathrm{Ct}\left(\text{short amplicon}\right)}$$

The value of 35 was chosen as the maximum number of Q‐PCR cycles the reaction underwent. A transformation of the differential amplicon was applied in order to have a theoretical maximal value of 1 (no degradation of RNA) and a theoretical minimal value close to 0 (totally degraded RNA).

### amoA *and* 16S rRNA *RT‐Q‐PCR*

For all degradation experiments, the Cts of the Bacterial *amoA* and the Bacterial *16S rRNA* was determined by Q‐PCR of the cDNA preparations. The *amoA* Q‐PCR was carried out in a 20 μl reaction volume composed of 10 μl EVAGreen Supermixes (SsoFast; Bio‐Rad), 0.4 μl of each primer (BacamoA‐1F and BacamoA‐2R) (10 μM each), 7.2 μl water and 2 μl of cDNA template (1/10 diluted). The Q‐PCR cycle was as follows: 95°C‐5 min, (95°C‐30s, 47°C‐30 s, 72°C‐1 min, 81°C‐1 s➔ plate read) × 40 cycles. Melt curve analysis was performed from 65°C to 95°C with 0.5°C increment every 5 s. *16S rRNA* cDNA targets were quantified in a 20 μl reaction volume composed of 10 μl Itaq Universal Probes Supermix (Bio‐Rad), 1.8 μl each primer (1369F and 1492r) (10 μM each), 0.4 μl probe (1389P) (10 μM), 5 μl water and 1 μl cDNA template (1/10 diluted). The Q‐PCR cycle was as follows: 95°C‐10 min, (95°C‐10s, 60°C‐30s) × 40 cycles and 40°C‐10 min. All primers are detailed in Table 1.

### *Illumina sequencing*

The qualitative effect of RNA degradation the community composition of the three bacterial genes (*amoA*, *glnA* and 16S rRNA) was determined by sequencing the amplicons generated from the cDNA preparations obtained after *RNAse I* degradation. For each PCR, amplification was carried out using the HotStartTaq PCR kit (Qiagen) in the following mix 25 μl volume: 19.8 μl water, 0.5 μl of each primer (10 μM each), 0.5 μl dNTPs (10 μM each), 0.2 μl HotStartTaq, 2.5 μl of 10x PCR buffer and 1 μl cDNA template (10^−1^ and 10^−3^ diluted for functional genes and 16S rRNA respectively). Primers used for sequencing are listed in Table 1 (Illumina adaptors were added at the 5′ end of the sequencing primers for PCR: 5′‐TCG TCG GCA GCG TCA GAT GTG TAT AAG AGA CAG (forward adaptor); 5′‐GTC TCG TGG GCT CGG AGA TGT GTA TAA GAG ACA G (reverse adaptor). The PCR cycles were as follows: *amoA*: 95°C‐15 min, (94°C‐30s, 55°C‐30s, 72°C‐30s) × 32 cycles and 72°C‐10 min final extension; *glnA*: 95°C‐15 min, (94°C‐30s, 55.6°C‐40s, 72°C‐40s) × 32 cycles and 72°C‐7 min final extension; 16S rRNA: 95°C‐15 min, (94°C‐45 s, 50°C‐30s, 72°C‐40s) × 25 cycles and 72°C‐10 min final extension. For each functional gene, three separate PCRs were carried out, using the same conditions, and pooled together for further processing.

PCR amplicons were cleaned using the AMPure XP beads kit following the manufacturer's recommendations. Illumina indexes were then attached using the Nextera XT Index Kit with the following PCR condition: 95°C‐15 min, (95°C‐30s, 55°C‐30s, 72°C‐30s) × 8 cycles and 72°C‐5 min. The resulting amplicons were purified using the AMPure XP beads kit and eluted in 25 μl water. After this step, some preparations were randomly chosen (2 per genes) and run on the Bioanalyser following the DNA 1000 Assay protocol (Agilent Technologies) to determine the average length of the amplicons and to check for the presence of unspecific products. Finally, DNA concentration was determined using fluorometric quantification method (Qubit) and molarity was calculated using the following equation:

(concentration in ng μl^−1^) × 10^6^ = (660 g mol^−1^ × average library size).

Libraries were pooled in equimolar amount and checked again on the Bioanalyser and the final library was sent to the Earlham Institute (Norwich Research Park, Norwich, UK) for Illumina MiSeq amplicon sequencing.

### *Bioinformatics*

#### *Construction of the reference databases*

The following sequences were downloaded (see Supporting Information Additional file 2): *amoA* sequences from Fungene (http://fungene.cme.msu.edu/) alongside NCBI sequences (*n* = 642); and bacterial *glnA* sequences (*n* = 1330) as FASTA files from Microbial Genome Database (http://mbgd.genome.ad.jp). For *amoA s*equences, the NCBI taxonomy was given in the FASTA headers whereas for *glnA* sequences, the MBGD Archive (http://mbgd.genome.ad.jp/htbin/view_arch.cgi) was used to download annotations (mbgd_2016_01) associated with the sequences, and a custom script was written to identify and tag the sequences with NCBI taxonomy. Subsequently, R's rentrez (Winter, 2017) package was used to get taxonomic information at different levels to generate a taxonomy file for *glnA* sequences. The FASTA file and the corresponding taxonomy file was then formatted to work with Qiime. For *16S rRNA* we used the SILVA SSU Ref NR database release v123.

#### *Processing of amplicon sequences*

Abundance tables were obtained by constructing operational taxonomic units (OTUs) as follows. Paired‐end reads were trimmed and filtered using Sickle v1.200 (Joshi and Sickle, 2011) by applying a sliding window approach and trimming regions where the average base quality drops below 20. Following this we apply a 10 bp length threshold to discard reads that fall below this length. We then used BayesHammer (Nikolenko *et al*., 2013) from the Spades v2.5.0 assembler to error correct the paired‐end reads followed by pandaseq v(2.4) with a minimum overlap of 20 bp to assemble the forward and reverse reads into a single sequence. The above choice of software was as a result of author's recent work (Schirmer *et al*., 2015; D'Amore *et al*., 2016) where it was shown that the above strategy of read trimming followed by error correction and overlapping reads reduces the substitution rates significantly. After having obtained the consensus sequences from each sample, the VSEARCH (v2.3.4) pipeline (all these steps are documented in https://github.com/torognes/vsearch/wiki/VSEARCH-pipeline) was used for OTU construction. The approach is as follows: the reads are pooled from different samples together and barcodes added to keep an account of the samples these reads originate from. Reads are then de‐replicated and sorted by decreasing abundance and singletons discarded. In the next step, the reads are clustered based on 97% similarity, followed by removing clusters that have chimeric models built from more abundant reads (‐‐uchime_denovo option in vsearch). A few chimeras may be missed, especially if they have parents that are absent from the reads or are present with very low abundance. Therefore, in the next step, we use a reference‐based chimera filtering step (‐‐uchime_ref option in vsearch) using a gold database (https://www.mothur.org/w/images/f/f1/Silva.gold.bacteria.zip) for *16S rRNA* sequences, and the above created reference databases for *glnA* and *amoA* genes. The original barcoded reads were matched against clean OTUs with 97% similarity to generate OTU tables (4108, 1691 and 55 OTU sequences for 16SrRNA, *glnA* and *amoA* respectively). The representative OTUs were then taxonomically classified using assign_taxonomy.py script from Qiime (Caporaso *et al*., 2010) against the reference databases. To find the phylogenetic distances between OTUs, we first multi sequence aligned the OTUs against each other using Mafft (Katoh *et al*., 2009) and then used FastTree v2.1.7 (Price *et al*., 2010) to generate the phylogenetic tree in NEWICK format. Finally, make_otu_table.py from Qiime workflow was employed to combine abundance table with taxonomy information to generate biome file for OTUs (Supporting Information Additional Files S5‐S7).

### *Statistical analysis*

All statistical analysis was carried out in R (R Development Core Team, 2008). For degradation experiments, RIN and *R*_amp_ values were compared between time points with one‐way ANOVA, when the ANOVA test was significant, differences between time points were investigated using Tuckey HSD post hoc test. For community analysis (including alpha and beta diversity analyses), the vegan package was used (Oksanen *et al*., 2005). To find OTUs that are significantly different between multiple conditions (Degradation), DESeqDataSetFromMatrix() function from DESeq2 (Love *et al*., 2014) package with the adjusted p‐value significance cut‐off of 0.05 and log2 fold change cut‐off of 2 was used. Vegan's adonis() was used for analysis of variance (henceforth referred to as PERMANOVA) using distance matrices (BrayCurtis/Unweighted Unifrac/Weighted Unifrac for gene sequences) i.e., partitioning distance matrices among sources of variation (Degradation). The scripts for above analysis can be found at http://userweb.eng.gla.ac.uk/umer.ijaz/#bioinformatics.

## Supporting information


**Additional file 1**

**Additional file 2**

**Additional file 3**

**Additional file 4**
**Fig. S1.** Effect of freeze/thaw on RNA integrity via RT‐Q‐PCR (A) and RIN versus R_amp_ (B). A) Effect of freeze/thaw on transcript quantification; Amp 1‐3: average Ct (*n* = 3) of one of the three possible *glnA* amplicons; *amoA*: average *amoA* Ct (*n* = 3) of the Bacterial *amoA* transcript; *16S rRNA*: average *16S rRNA* Ct (*n* = 3) of the bacterial *16S rRNA* transcript. Effect of RNA degradation on R_amp_ index is presented in Fig. B. For comparison, RIN values were also plotted.
**Fig. S2.** Effect of storage on RNA integrity via RT‐Q‐PCR (A) and RIN versus R_amp_ (B). A) Effect of storage on transcript quantification; Amp 1‐3: average Ct (*n* = 3) of one of the three possible *glnA* amplicons; *amoA*: average *amoA* Ct (*n* = 3) of the Bacterial *amoA* transcript; *16S rRNA*: average *16S rRNA* Ct (*n* = 3) of the bacterial *16S rRNA* transcript. Effect of RNA degradation on R_amp_ index is presented in Fig. B. For comparison, RIN values were also plotted.
**Fig. S3.** Evolutionary relationships of the 84 bacterial glnA genes used to design new primers. The evolutionary history was inferred using the Neighbor‐Joining method (Saitou and Nei, 1987). The optimal tree with the sum of branch length = 10.74788158 is shown. The tree is drawn to scale, with branch lengths in the same units as those of the evolutionary distances used to infer the phylogenetic tree. The evolutionary distances were computed using the Maximum Composite Likelihood method (Tamura et al., 2004) and are in the units of the number of base substitutions per site. The analysis involved 84 nucleotide sequences. Codon positions included were 1st+2nd+3rd+Noncoding. All positions containing gaps and missing data were eliminated. There were a total of 690 positions in the final data set. Evolutionary analyses were conducted in MEGA7 (Kumar et al., 2015).
**Fig. S4.** Normalisation of amoA and 16s rRNA RT‐qPCR results to RNA integrity. The correction of the Cts was done by assuming linear relationship between the change in Cts and the change in Ramp index along the degradation gradient i.e. change in Ct = α × change in Ramp. Cts corrected for RNA integrity was then calculated as follows: corrected Ct = Ct – (α × (RamptX ‐ Rampt0)) with RamptX corresponding to the Ramp at a degradation point X and Rampt0 corresponding to the Ramp at the initial point. Both Ramp 380/120 and Ramp 380/170 were used to calculate the correction coefficient α.
**Table S1.** Summary of the regression coefficients associated with the equation: change in Ct = f(change in *R*_amp_). The coefficients are calculated assuming a linear relationship.

**Additional file 5**

**Additional file 6**

**Additional file 7**
