# Supplementary material for: Differential ratio amplicons (R amp) for the evaluation of RNA integrity extracted from complex environmental samples
Source: Environ Microbiol. 2019 Feb 12;21(2):827–44. doi: 10.1111/1462-2920.14516 (PMC6392129; doi:10.1111/1462-2920.14516)
Supplement: Supplementary file 2 — Additional file 2 [file EMI-21-827-s002.docx]

**Supplementary Section:**

**Steps to generate glnA database and taxonomy file to work with Qiime**

To make a database from Microbial Genome Database (MGBD) <http://mbgd.genome.ad.jp>, we need to follow the following steps:

**Step 1**:
From MBDG downloaded sequences for a glnA bacterial sequences and save it in a FASTA file called glnA.fa. Then generate a new FASTA file that only has organism names
awk '/>/{gsub("^>","",$0);gsub(":.*","",$0);$0=">F"++i"_"$0}1' glnA.fa > glnA_symbol.fa

**Step 2**: Get the organism IDs and store them in IDs.csv
awk '/>/{a=$0;sub("^>F[0-9]+_","",$0); print a","$0}' glnA_symbol.fa > IDs.csv

**Step 3**: Get mapping to NCBI gids. For this purpose, download [http://mbgd.genome.ad.jp/dist/mbgd_2016-01/mbgd_2016-01_gene.gz](https://mail.campus.gla.ac.uk/owa/redir.aspx?C=UCTEpL8s21xI0M9wj_EZJFOnw05_cPpNFat8hHE1SnOWWHLkz5rVCA..&URL=http%3a%2f%2fmbgd.genome.ad.jp%2fdist%2fmbgd_2016-01%2fmbgd_2016-01_gene.gz)  from [http://mbgd.genome.ad.jp/htbin/view_arch.cgi](https://mail.campus.gla.ac.uk/owa/redir.aspx?C=004rzVZD_WpbLh1_Z8Fqbaa2ywOjmUb3FJ7Dxbr1ngaWWHLkz5rVCA..&URL=http%3a%2f%2fmbgd.genome.ad.jp%2fhtbin%2fview_arch.cgi) 
awk -F"\t" 'BEGIN{while((getline k < "IDs.csv")>0){split(k,a,",");m[a[1]]=a[2];o[a[2]]=1}}{if(o[$1]){if(length($12)>1){o[$1]=$12}}}END{for(i in m){print i","o[m[i]]}}' mbgd_2016-01_gene > mapping.csv

**Step 4**: Use the mapping file to change the FASTA headers
awk 'BEGIN{while((getline k < "mapping.csv")>0){split(k,a,",");m[a[1]]=a[2]}}/^>/{b=$1;gsub("_.*","",b);$0=b"_"m[$1]}1' glnA_symbol.fa > glnA_reference_db.fa

**Step 5**: Extract a comma-delimited IDs along with GIDs
awk -F"_" 'BEGIN{print "ID,GID"}/^>/{gsub(">","",$0);print $1","$2}' glnA_reference_db.fa > glnA_reference_db_id.csv

**Steps to generate amoA database and taxonomy file to work with Qiime**

To make a database from Fungene (<http://fungene.cme.msu.edu/> ), download amoA sequences to amoA.fa.

**Step 1**: Format so that we have >ID_GID
awk '/^>/{gsub(" .*","",$0); gsub(">","",$0); $0=">F"++i"_"$0}1' amoA.fa > amoA_reference_db.fa

**Step 2**: Extract a comma-delimited IDs along with GIDs so that we can use an R package (taxize) to get the complete taxonomy out
awk -F"_" 'BEGIN{print "ID,GID"}/^>/{gsub(">","",$0);print $1","$2}' amoA_reference_db.fa > amoA_reference_db_id.csv

**R script to generate taxonomy files (common to both glnA and amoA)**

Use glnA_reference_db_id.csv/amoA_reference_db_id.csv to generate glnA_reference_db_id.tax/ amoA_reference_db_id.tax.
library(rentrez)
#Load the mapping table up
mapping_table<-read.csv("**glnA_reference_db_id.csv**",row.names=1,header=T)
#extract gids
gids<-mapping_table$GID
taxa_levels<-NULL
for(i in seq(1:length(gids))){
  print(paste("Processing",i,"/",length(gids)))
  tmp<-tryCatch(paste(XML::xpathSApply(entrez_fetch(db="taxonomy",id=entrez_summary(db="nucleotide", id=gids[i])$taxid,rettype="xml", parsed=TRUE), "//LineageEx/Taxon/ScientificName", XML::xmlValue),collapse=";"),error=function(e) "")
  if(is.null(taxa_levels)){taxa_levels<-tmp}else{taxa_levels<-c(taxa_levels,tmp)}
}
taxa_levels[taxa_levels==""]<-"cellular organisms;unassigned"
data_to_write<-data.frame(ID=paste(rownames(mapping_table),mapping_table[,1],sep="_"),Taxa=taxa_levels)
write.table(data_to_write,"**glnA_reference_db.tax**",sep="\t",row.names=F,col.names=F,quote=F)
