# Supplementary material for: Differential ratio amplicons (R amp) for the evaluation of RNA integrity extracted from complex environmental samples
Source: Environ Microbiol. 2019 Feb 12;21(2):827–44. doi: 10.1111/1462-2920.14516 (PMC6392129; doi:10.1111/1462-2920.14516)
Supplement: Supplementary file 4 — Additional file 4 Fig. S1. Effect of freeze/thaw on RNA integrity via RT‐Q‐PCR (A) and RIN versus Ramp (B). A) Effect of freeze/thaw on transcript quantification; Amp 1‐3: average Ct (n = 3) of one of the three possible glnA amplicons; amoA: average amoA Ct (n = 3) of the Bacterial amoA transcript; 16S rRNA: average 16S rRNA Ct (n = 3) of the bacterial 16S rRNA transcript. Effect of RNA degradation on Ramp index is presented in Fig. B. For comparison, RIN values were also plotted. Fig. S2. Effect of storage on RNA integrity via RT‐Q‐PCR (A) and RIN versus Ramp (B). A) Effect of storage on transcript quantification; Amp 1‐3: average Ct (n = 3) of one of the three possible glnA amplicons; amoA: average amoA Ct (n = 3) of the Bacterial amoA transcript; 16S rRNA: average 16S rRNA Ct (n = 3) of the bacterial 16S rRNA transcript. Effect of RNA degradation on Ramp index is presented in Fig. B. For comparison, RIN values were also plotted. Fig. S3. Evolutionary relationships of the 84 bacterial glnA genes used to design new primers. The evolutionary history was inferred using the Neighbor‐Joining method (Saitou and Nei, 1987). The optimal tree with the sum of branch length = 10.74788158 is shown. The tree is drawn to scale, with branch lengths in the same units as those of the evolutionary distances used to infer the phylogenetic tree. The evolutionary distances were computed using the Maximum Composite Likelihood method (Tamura et al., 2004) and are in the units of the number of base substitutions per site. The analysis involved 84 nucleotide sequences. Codon positions included were 1st+2nd+3rd+Noncoding. All positions containing gaps and missing data were eliminated. There were a total of 690 positions in the final data set. Evolutionary analyses were conducted in MEGA7 (Kumar et al., 2015). Fig. S4. Normalisation of amoA and 16s rRNA RT‐qPCR results to RNA integrity. The correction of the Cts was done by assuming linear relationship between the change in Cts and the chan [file EMI-21-827-s004.docx]

**B**

**A**

**Fig S.1. Effect of freeze/thaw on RNA integrity via RT-Q-PCR (A) and RIN versus R_amp_ (B).** A) Effect of freeze/thaw on transcript quantification; Amp 1-3: average Ct (n=3) of one of the three possible *glnA* amplicons; *amoA*: average *amoA* Ct (n=3) of the Bacterial *amoA* transcript; *16S rRNA*: average *16S rRNA* Ct (n=3) of the bacterial *16S rRNA* transcript. Effect of RNA degradation on R_amp_ index is presented in Fig. B. For comparison, RIN values were also plotted.

**Fig S.2. Effect of storage on RNA integrity via RT-Q-PCR (A) and RIN versus R_amp_ (B).** A) Effect of storage on transcript quantification; Amp 1-3: average Ct (n=3) of one of the three possible *glnA* amplicons; *amoA*: average *amoA* Ct (n=3) of the Bacterial *amoA* transcript; *16S rRNA*: average *16S rRNA* Ct (n=3) of the bacterial *16S rRNA* transcript. Effect of RNA degradation on R_amp_ index is presented in Fig. B. For comparison, RIN values were also plotted.

**Figure S.3. Evolutionary relationships of the 84 bacterial *glnA* genes used to design new primers**

The evolutionary history was inferred using the Neighbor-Joining method (Saitou & Nei, 1987). The optimal tree with the sum of branch length = 10.74788158 is shown. The tree is drawn to scale, with branch lengths in the same units as those of the evolutionary distances used to infer the phylogenetic tree. The evolutionary distances were computed using the Maximum Composite Likelihood method (Tamura et al., 2004) and are in the units of the number of base substitutions per site. The analysis involved 84 nucleotide sequences. Codon positions included were 1st+2nd+3rd+Noncoding. All positions containing gaps and missing data were eliminated. There were a total of 690 positions in the final dataset. Evolutionary analyses were conducted in MEGA7 (Kumar et al., 2015).

**8**

**7**

**6**

**5**

**4**

**3**

**2**

**1**


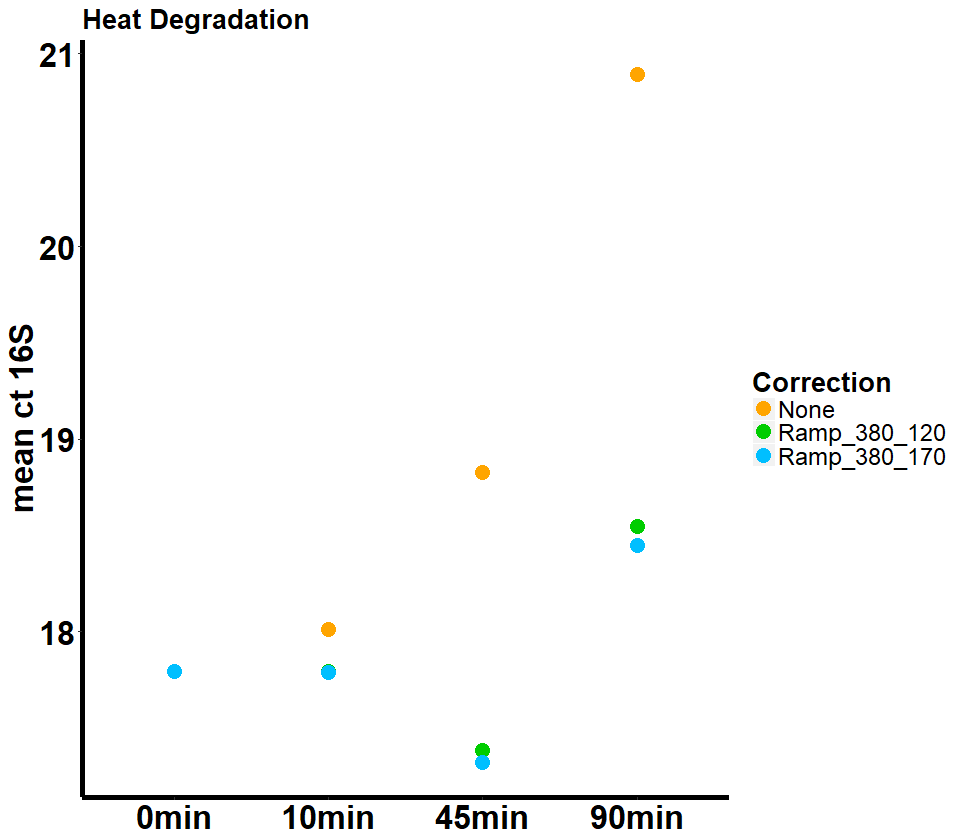

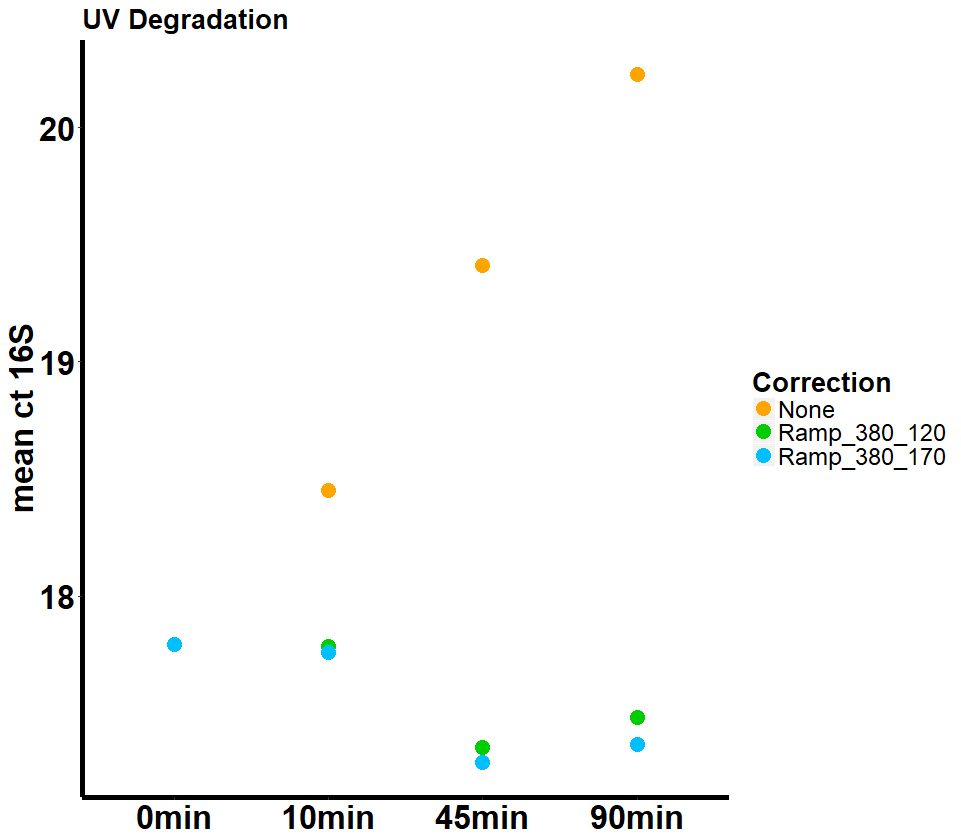

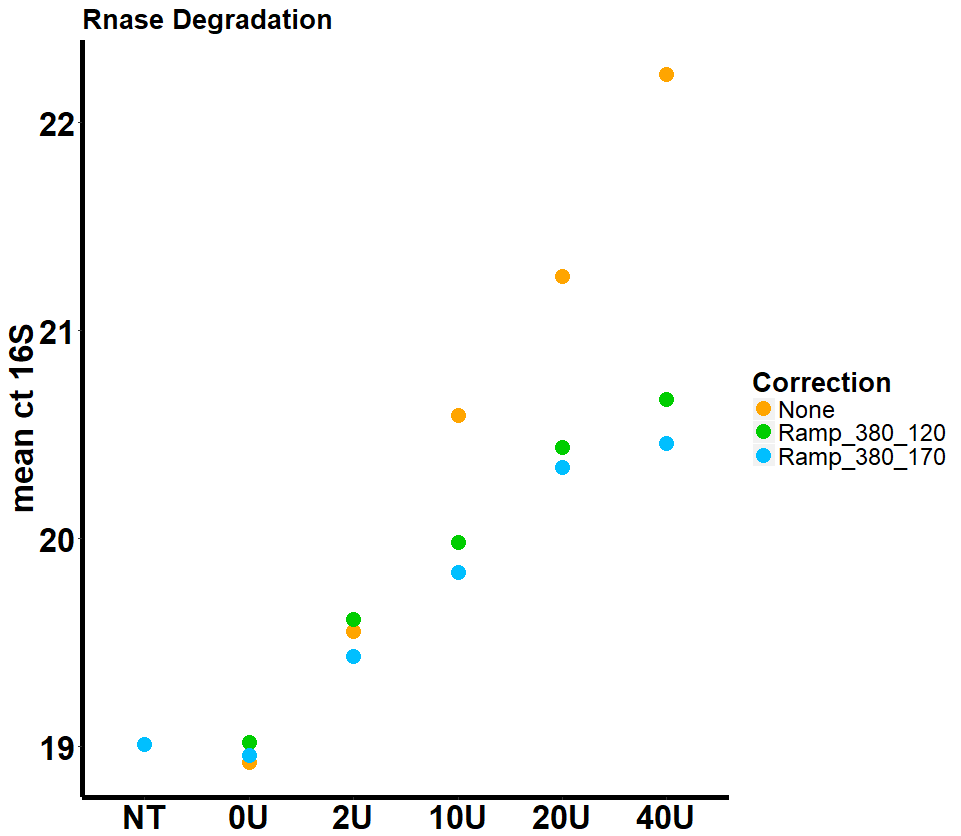

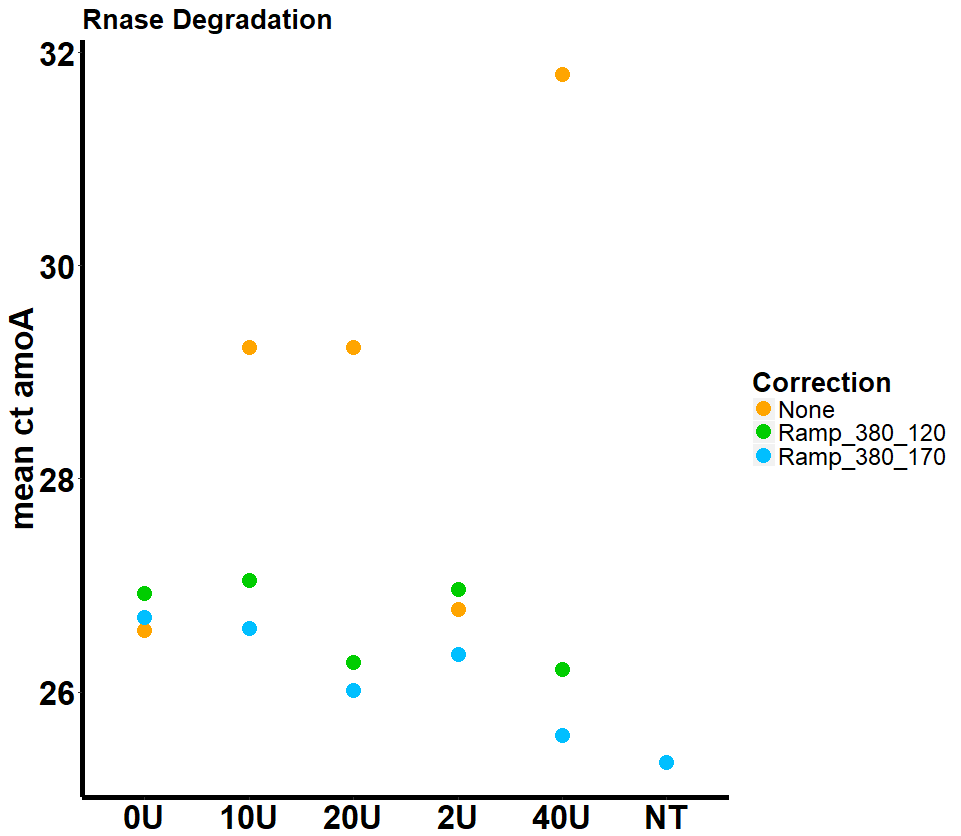

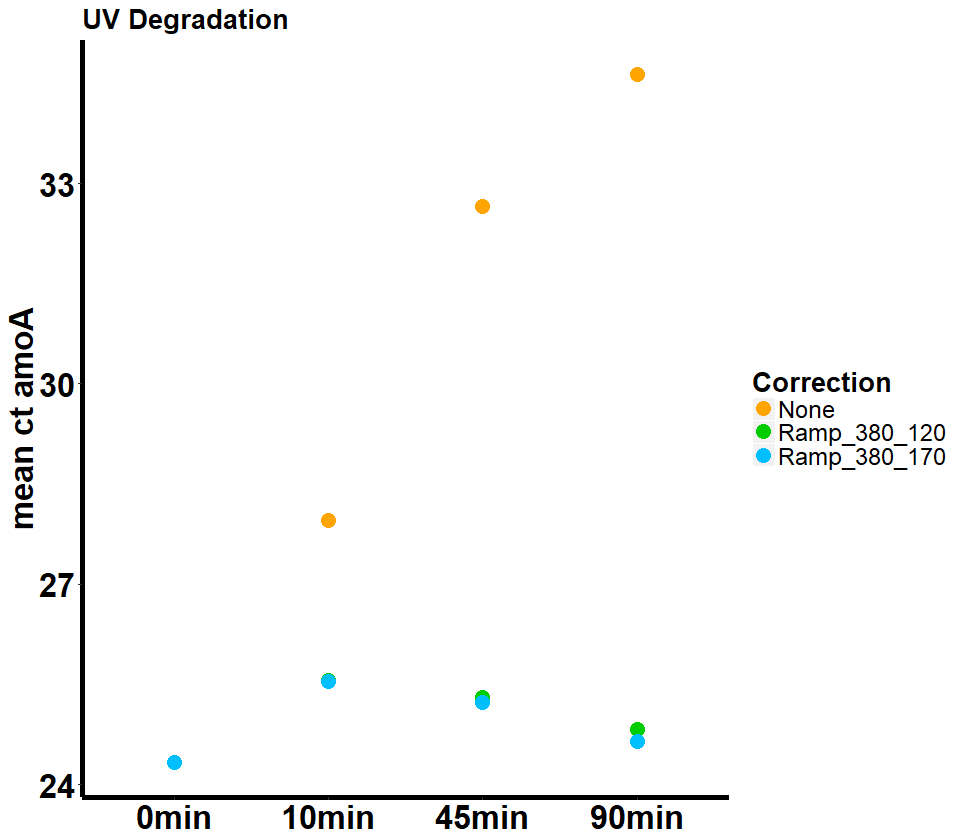

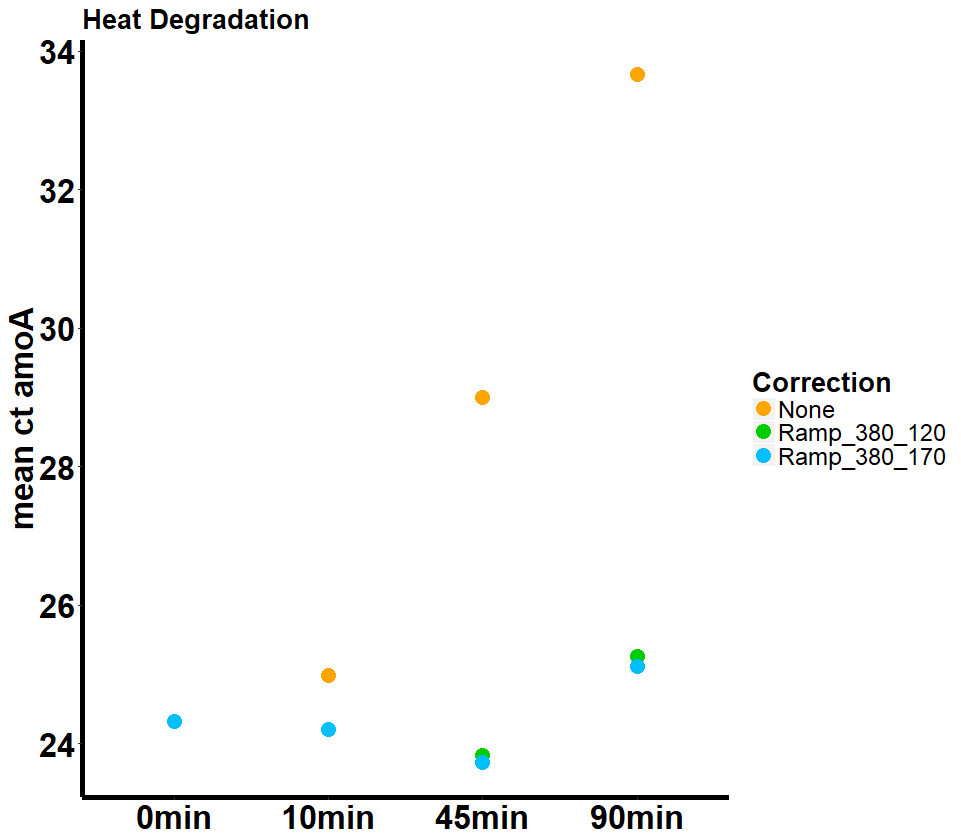


**Figure S.4. Normalisation of *amoA* and *16s rRNA* RT-qPCR results to RNA integrity.** The correction of the Cts was done by assuming linear relationship between the change in Cts and the change in R_amp_ index along the degradation gradient *i.e.* change in Ct = α x change in R_amp_. Cts corrected for RNA integrity was then calculated as follows:

corrected Ct = Ct – (α x (R_amp_t_X_ - R_amp_t_0_))

with R_amp_t_X_ corresponding to the R_amp_ at a degradation point X and R_amp_t_0_ corresponding to the R_amp_ at the initial point. Both R_amp_ 380/120 and R_amp_ 380/170 were used to calculate the correction coefficient α.

**Table S.1. Summary of the regression coefficients associated with the equation: change in Ct = f(change in R_amp_).** The coefficients are calculated assuming a linear relationship.

| **Gene** | **Degradation** | **R_amp_ 380/120** | | **R_amp_ 380/170** | |
| --- | --- | --- | --- | --- | --- |
|  |  | **Slope (**α**) (p-value)** | **Adjusted R-squared** | **Slope (**α**) (p-value)** | **Adjusted R-squared** |
| ***amoA*** | Heat | 12.75 (0.007) | 0.98 | 14.31 (0.007) | 0.98 |
|  | UV | 12.07 (0.007) | 0.98 | 13.56 (0.007) | 0.98 |
|  | RNase | 11.34 (0.002) | 0.90 | 12.50 (0.0009) | 0.94 |
| ***16S rRNA*** | Heat | 4.03 (0.0445) | 0.87 | 4.52 (0.0441) | 0.87 |
|  | UV | 2.78 (0.0050) | 0.99 | 3.12 (0.0045) | 0.99 |
|  | RNase | 6.50 (0.0007) | 0.95 | 7.09 (0.0004) | 0.96 |
